# Measurement-device-independent quantum key distribution with leaky sources

**DOI:** 10.1038/s41598-021-81003-2

**Published:** 2021-01-18

**Authors:** Weilong Wang, Kiyoshi Tamaki, Marcos Curty

**Affiliations:** 1 EI Telecomunicación, Department of Signal Theory and Communications, University of Vigo, 36310 Vigo, Spain; 2 State Key Laboratory of Mathematical Engineering and Advanced Computing, Zhengzhou, 450001 Henan China; 3 Henan Key Laboratory of Network Cryptography Technology, Zhengzhou, 450001 Henan China; 4 Faculty of Engineering, University of Toyama, Gofuku 3190, Toyama, 930-8555 Japan

**Keywords:** Quantum information, Qubits

## Abstract

Measurement-device-independent quantum key distribution (MDI-QKD) can remove all detection side-channels from quantum communication systems. The security proofs require, however, that certain assumptions on the sources are satisfied. This includes, for instance, the requirement that there is no information leakage from the transmitters of the senders, which unfortunately is very difficult to guarantee in practice. In this paper we relax this unrealistic assumption by presenting a general formalism to prove the security of MDI-QKD with leaky sources. With this formalism, we analyze the finite-key security of two prominent MDI-QKD schemes—a symmetric three-intensity decoy-state MDI-QKD protocol and a four-intensity decoy-state MDI-QKD protocol—and determine their robustness against information leakage from both the intensity modulator and the phase modulator of the transmitters. Our work shows that MDI-QKD is feasible within a reasonable time frame of signal transmission given that the sources are sufficiently isolated. Thus, it provides an essential reference for experimentalists to ensure the security of implementations of MDI-QKD in the presence of information leakage.

## Introduction

In theory, quantum key distribution (QKD)^1–4^ provides an information-theoretically secure way to distribute secret keys between two distant parties (commonly known as Alice and Bob). In practice, however, this is not the case. This is so because real devices do not typically conform to the requirements imposed by the security proofs. Indeed, various types of quantum hacking attacks have been proposed and experimentally demonstrated recently, which exploit device’ imperfections in practical QKD systems^4^. To tackle these implementation security loopholes, many efforts have been made, among which device-independent (DI) QKD^5–7^ and measurement-device-independent (MDI) QKD^8^ are two prominent approaches. The security of DI-QKD relies on the violation of a Bell inequality^9,10^ and no knowledge about the inner working of the quantum apparatuses is needed given that the apparatuses are ‘honest’^11^, i.e., given that they follow the prescriptions of the protocol and not those of Eve. DI-QKD is, however, difficult to implement experimentally with current technology, especially for long distances^12–14^. On the other hand, thanks to its feasibility, MDI-QKD has attracted great attention and has been widely experimentally demonstrated in recent years^15–22^. In terms of security, MDI-QKD closes all side-channels in the detection unit, which significantly simplifies the path towards achieving implementation security in QKD, as now one only needs to secure the source. MDI-QKD requires, however, that certain assumptions on the sources are satisfied.

A common assumption is that Alice’s and Bob’s transmitters do not leak any unwanted information out of their security zones. Inspired by the results introduced in^23–25^, which study the information leakage problem in standard decoy-state QKD systems, here we relax such an unrealistic requirement and perform a finite-key security analysis of MDI-QKD with leaky sources. In particular, we focus on information leakage from two main apparatuses within the transmitters, the intensity modulator (IM), which is used to generate decoy states, and the phase modulator (PM), which is used to encode the basis and bit information. For instance, such information leakage might be due to a Trojan-horse attack (THA)^26^ performed by Eve. In this framework, we evaluate the security of two prominent MDI-QKD protocols: the symmetric three-intensity decoy-state MDI-QKD scheme^27^, and the efficient four-intensity decoy-state MDI-QKD protocol introduced in^28^, which has recently been implemented over a distance of 404 km^20^. As expected, our results show that MDI-QKD is more sensitive to information leakage than standard decoy-state QKD. Still, we show that MDI-QKD is feasible within a reasonable time frame of signal transmission given that Alice’s and Bob’s sources are sufficiently isolated.

## Methods

### The symmetric three-intensity decoy-state MDI-QKD protocol

We begin by describing the specific steps of the symmetric three-intensity decoy-state MDI-QKD protocol. Here, we consider a sifting strategy which protects the protocol against the sifting attack^29^. This is so because the total number of pulses sent by Alice and Bob is fixed a priori and, moreover, the termination condition is basis independent^30^. The assumptions that we make on the users’ devices in the absence of information leakage can be found in the Supplementary Information 1. The steps of the protocol are as follows:

1. *State preparation:* The first two steps of the protocol are repeated *N* times, where *N* is a prefixed number. In each round, Alice and Bob select a basis $\chi \in \{\text{Z},~\text{X}\}$ with probabilities $p_{\text{Z}}$ and $p_{\text{X}}=1-p_{\text{Z}}$, and select an intensity setting $\gamma ^{j_{\text{A}}}$ and $\gamma ^{j_{\text{B}}}$ with $j_{\text{A}},~j_{\text{B}} \in \{\text{s,v,w}\}$, with probability $p_{j_{\text{A}}}$ and $p_{j_{\text{B}}}$, respectively. Afterwards, each of them encodes a random bit in a phase-randomized WCP of the chosen intensity in the chosen basis by using, for instance, the polarization encoding scheme employed in Ref.^8^ and sends it to the untrusted relay via the quantum channel. Note that our analysis is valid for any other encoding scheme.
2. *Measurement:* The untrusted relay is supposed to perform a Bell state measurement (BSM) on the states received from Alice and Bob and then record the measurement outcomes. For concreteness, below we shall assume that the untrusted relay uses the BSM introduced in Ref.^8^, which is based on linear optical elements and can distinguish two Bell states. In reality, however, the relay can behave as Eve decides.
3. *Announcement of the measurement outcomes and random data post-selection:* Once the *N* rounds of steps 1 and 2 have finished, the relay announces in which rounds he obtained successful measurements together with the corresponding measurement outcomes. For each successful measurement event, Alice selects a fictitious basis ${\text{Z}}_{\text{A}_\text{{c}}}$ or $\text{X}_{\text{A}_{\text{c}}}$ with probability $p_{\text{Z}_{{\text{A}}_{\text{c}}}}$ and $p_{\text{X}_{\text{A}_\text{c}}}=1-p_{\text{Z}_{\text{A}_{\text{c}}}}$, respectively, and then she announces her fictitious basis choices.
4. *Sifting:* If Alice’s choice is the $\text{X}_{\text{A}_{\text{c}}}$ basis, Bob announces his state preparation basis choice but Alice does not announce hers and then they discard the corresponding data. If Alice’s choice is the $\text{Z}_{\text{A}_\text{c}}$ basis, both Alice and Bob announce their state preparation basis choices as well as their intensity settings. We denote by $Z^{j_{\text{A}}j_{\text{B}}}$ ($X^{j_{\text{A}}j_{\text{B}}}$) the set of indexes that identify the successful measurement events when Alice and Bob select the intensity settings $\gamma ^{j_{\text{A}}}$ and $\gamma ^{j_{\text{B}}}$, respectively, Alice chooses the fictitious basis $\text{Z}_{\text{A}_\text{c}}$, and both of them select the Z (X) basis. If the sifting conditions $| Z^{j_{\text{A}}j_{\text{B}}} | \ge N^{j_{\text{A}}j_{\text{B}}}_{\text{Z}}$ and $| X^{j_{\text{A}}j_{\text{B}}} |\ge N^{j_{\text{A}}j_{\text{B}}}_{\text{X}}$ are satisfied for all ${j_{\text{A}},~j_{\text{B}}}\in \{\text{s,v,w}\}$, where $N^{j_{\text{A}}j_{\text{B}}}_{\text{Z}}$ and $N^{j_{\text{A}}j_{\text{B}}}_{\text{X}}$ are prefixed threshold values, Alice and Bob proceed to execute the next steps of the protocol. If the sifting conditions are not satisfied, the protocol aborts.
5. *Parameter estimation:* Alice and Bob estimate a lower bound, which we denote by $N^{L}_{{\text{click,00},\text{ss}}|\text{Z}}$ ($N^{L}_{{\text{click,11},\text{ss}}|\text{Z}}$), on the number of successful measurement events in the sifted key data set ${Z}^{\text{ss}}$, in which both of them sent vacuum (single-photon) pulses. Also they use all the data in the sets ${Z}^{k_{\text{A}}k_{\text{B}}}$ and ${X}^{j_{\text{A}}j_{\text{B}}}$, except that in the set $Z^\text{ss}$, to estimate an upper bound on the single-photon phase error rate in the sifted key data set ${Z}^\text{ss}$, which we denote by $e^\text{U}_\text{ph}$.
6. *Information reconciliation and privacy amplification:* Alice and Bob perform an error correction step for a predetermined quantum bit error rate (QBER), which we denote by $E_{\text{Z}}^\text{ss}$. Then Alice computes a hash of the sifted key data in $Z^{\text{ss}}$ by using a random universal$_{\text{2}}$ hash function^31^ and sends Bob the hash value together with the hash function. Bob uses the hash function to compute a hash of his corrected sifted key data and checks if the hash value coincides with that of Alice. If both hash values coincide, this error verification step guarantees that they share identical keys after error correction except for an exponentially small probability. Moreover, if this step succeeds, then they perform a privacy amplification step by applying a random universal$_{\text{2}}$ hash function to distill the final secret key.

Note that the sifting condition in Step 4 of the above protocol is only for data processing, and it is not related to the termination of the quantum communication steps, i.e., Steps 1 and 2, which is basis independent. Therefore, as indicated above, the protocol is secure against the sifting attack^30^.

### Parameter estimation method for the three-intensity protocol with leaky sources

In this section we briefly explain the general idea of our method to estimate the relevant parameters that are required to evaluate the secret key rate formula in the presence of information leakage. For concreteness, we consider the security analysis introduced in^32^, which provides a lower bound on the secret key length, $\ell$, given by

1 $$\begin{aligned} \ell \ge N^{L}_{{\text{click,00},\text{ss}}|\text{Z}} + N^{L}_{{\text{click,11}, \text{ss}}|\text{Z}} \left[ 1 - H\left( {e^{\text{U}}_{\text{ph}}}\right) \right] - leak{_{\text{EC}}} - {\log _2}\frac{2}{{{\varepsilon ^2 _{{\text{sec}}}} - \varepsilon }} - {\log _2}\frac{2}{{{\varepsilon _{{\text{cor}}}}}} , \end{aligned}$$

where $H( x ) = - x{\log _2}( x ) - ( {1 - x} ){\log _2}( {1 - x} )$ is the binary Shannon entropy function. The parameter $leak_{\text{EC}}$ is the amount of syndrome information declared by Alice in the error correction step of the protocol, given by $leak_{\text{EC}}=|Z^\text{ss}|f_{\text{EC}}H(E_{\text{Z}}^{\text{ss}})$ for simplicity, where the parameter $f_{\text{EC}}$ is the efficiency of the error correction code. The quantities $\varepsilon _{{\text{sec}}}$ and $\varepsilon _{{\text{cor}}}$ are the secrecy and correctness parameters of the protocol, respectively, and $\varepsilon \le 1 - {\varepsilon _{\text{Z,00}}}{\varepsilon _{\text{Z,11}}}{\varepsilon _{\text{ph,11}}}$ with $\varepsilon _{\text{Z,00}}$, $\varepsilon _{\text{Z,11}}$ and $\varepsilon _{\text{ph,11}}$ being defined as the success probabilities when estimating the quantities $N^{L}_{{\text{click,00},\text{ss}}|\text{Z}}$, $N^{L}_{{\text{click,11}, \text{ss}}|\text{Z}}$ and $e^{\text{U}}_{\text{ph}}$, respectively. In other words, $\varepsilon$ denotes the failure probability that at least one of the estimations of $N^{L}_{{\text{click,00},\text{ss}}|\text{Z}}$, $N^{L}_{{\text{click,11}, \text{ss}}|\text{Z}}$ and $e^{\text{U}}_{\text{ph}}$ is incorrect.

**Figure 1:**
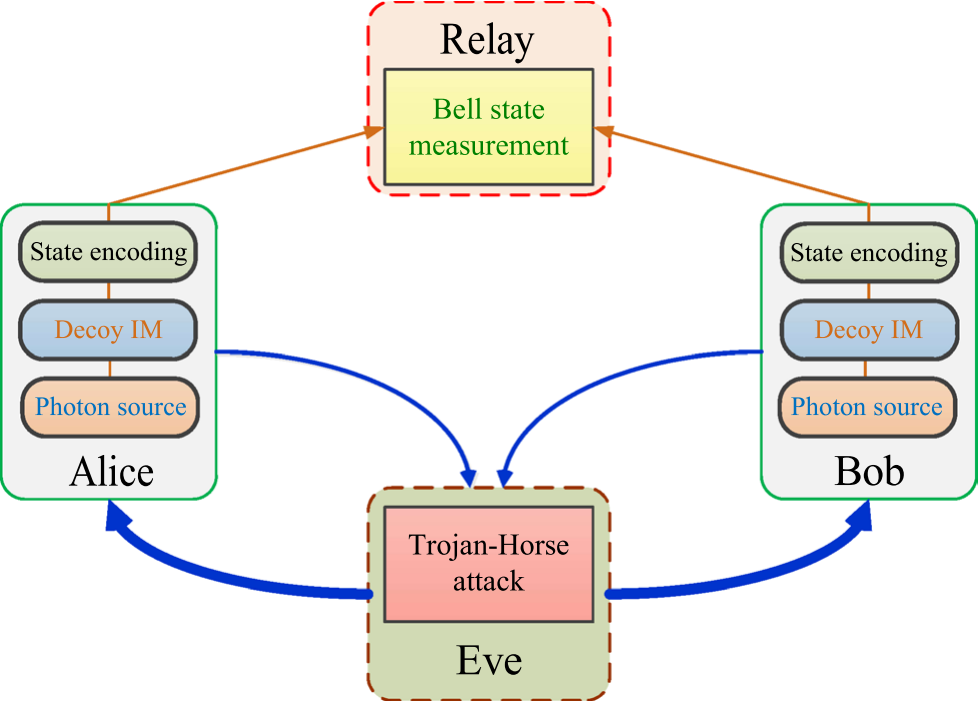
Each of Alice and Bob uses a photon source to prepare phase-randomised WCPs. Decoy states are generated by means of an intensity modulator (Decoy IM). The bit and basis information of the pulses are encoded with a state encoding setup (Encoding PM). The relay is supposed to perform a Bell state measurement on the incoming pulse pairs. In a THA, Eve actively sends bright light pulses (thick blue arrows) into Alice’s and Bob’s devices to trigger the emission of side-channel signals. Then, Eve measures the back-reflected light (thin blue arrows) to extract information about Alice’s and Bob’s internal settings. Note that since the relay is untrusted (i.e., it can be even Eve), in this figure we consider that it is the relay who performs the THA.

In the following we explain how to estimate the quantities $N^{L}_{{\text{click,00},\, \text{ss}}|{\text{Z}}}$, $N^{L}_{{\text{click,11}, \text{ss}}|\text{Z}}$ and $e^{\text{U}}_{\text{ph}}$ in the presence of information leakage. The detailed calculations can be found in the Supplementary Information 1. For concreteness, we shall assume that the information leakage is due to a THA performed by an active Eve. In this THA against the MDI-QKD system, Eve separately sends bright light into Alice’s and Bob’s devices and then measures the back-reflected light. In so doing, she can obtain partial information about Alice’s and Bob’s internal settings for each experimental trial. See Fig. 1 for an illustration of Eve’s THA. We remark, however, that our method is general and can be applied to analyze passive information leakage scenarios as well.

#### THA against the intensity modulator

Here, we briefly indicate the key ideas to analyze a THA targeted against the intensity modulator (IM), which is used to generate decoy states. The detailed calculations can be found in the Supplementary Information 1. In particular, we first consider an asymptotic scenario where Alice and Bob send an infinite number of pulses. In this scenario, we mainly apply the trace distance argument^24,25,33^ to relate the detection and error events arising from different intensity settings of Alice and Bob and obtain some linear relations between them. Then, by applying Azuma’s inequality^34^, the relations can be extended to the realistic regime where Alice and Bob send a finite number (*N*) of pulses. Finally, given the constraints provided by the mathematical relations obtained in the previous step, the relevant parameters which are needed to evaluate Eq. (1) can be estimated by using, for instance, linear programming techniques^35^.

#### THA against the phase modulator

A THA against the phase modulator (PM) might render Alice’s and Bob’s output states (which now also contain Eve’s systems due to the THA) *basis dependent*. As a result, Eve might be able to learn partial information about Alice’s and Bob’s basis and bit value choices each given time. The security of the standard BB84 protocol with a basis-dependent flaw has been analyzed in a previous work^36^ by using the idea of a quantum coin^37,38^. This idea was then generalized to phase encoding schemes for MDI-QKD where both Alice and Bob have basis-dependent flaws^39^. Here, to estimate the phase error rate in the presence of a THA against the PM, we apply the method introduced in Ref^39^ to our protocol.

More specifically, to simplify the analysis, we first consider a scenario where Alice’s and Bob’s light sources are both ideal single-photon sources. Also, we assume that Alice’s and Bob’s basis choices are random and do not depend on the IM or on the state of previous emitted pulses. Precisely, we consider a virtual entanglement scenario where each of Alice and Bob prepares a bipartite entangled state and then measures one of the two systems to actually prepare the states that are sent to the untrusted relay. We then apply the Bloch sphere bound^40^ to this fictitious scenario and obtain the mathematical relation between the expected number of events, which contains the expected number of phase errors in the asymptotic limit. Next, we extend it to the finite-key regime by using Azuma’s inequality, which contains the actual number of phase errors. Finally, the upper bound on the number of phase errors can be numerically estimated by simply using the optimization toolbox of Matlab, and thus we obtain the upper bound on the phase error rate. More details can be found in the Supplementary Information 1.

### The four-intensity decoy-state MDI-QKD protocol

We now consider the four-intensity decoy-state MDI-QKD protocol introduced in^28^, which has been recently implemented over a distance of 404 km^20^. In this protocol, each of Alice and Bob uses one intensity setting $\gamma ^{\text{s}}$ for the Z basis states, and three intensity settings $\gamma ^{\text{v}}$, $\gamma ^{\text{w}}$ and $\gamma ^{\text{0}}=0$ for the X basis states. This is motivated by the fact that in order to increase the number of single-photon pulses emitted in the Z basis used for key generation, the intensity of the signal states, $\gamma _{\text{s}}$, needs to be close to one, while in order to have a tight estimation of the relevant parameters, the intensities in the X basis used for parameter estimation need to be much weaker. With the four-intensity decoy-state MDI-QKD protocol, one can optimize the intensities for key generation and parameter estimation independently. The probabilities to select the corresponding intensities are $p_{\text{s}}$, $p_{\text{v}}$, $p_{\text{w}}$ and $p_{\text{0}}$, respectively, with $p_{\text{s}}+p_{\text{v}}+p_{\text{w}}+p_{\text{0}}=1$. Note that the probability to choose the Z basis is now $p_{\text{Z}}=p_{\text{s}}$ and the probability to choose the X basis is given by $p_{\text{X}}=p_{\text{v}}+p_{\text{w}}+p_{\text{0}}$.

### Parameter estimation method for the four-intensity protocol with leaky sources

The security analysis of this protocol against information leakage from the IM and the PM is slightly different from that in the previous section. This is because of the following. Since now the intensity setting in the Z basis is unique and it is typically different from the intensity settings in the X basis, by analyzing the information leakage from the IM Eve can also learn partial information about the users’ basis choices. Similarly, by analyzing the information leakage from the PM Eve can learn partial information about the users’ intensity settings as well. That is, the information leakage from the IM and the PM of each user is now correlated. Fortunately, a general procedure to estimate the relevant parameters has already been briefly introduced in Ref^24^. Here, we adapt it to the scenario of the four-intensity decoy-state MDI-QKD protocol.

Note that, in general, when the IM and the PM are correlated, the yields associated with different photon number states can also depend on the bit value^24^. However, for simplicity, in the model above we assume that the back-reflected light does not carry information about the bit value but only about the basis. The specific calculations for the relevant parameters to evaluate Eq. (1) can be found in the Supplementary Information 1.

## Results

The secret key rates in the presence of information leakage can be simulated given the security analysis summarized above. In this section, we show and compare the results for the three-intensity and four-intensity protocols.

### Simulation results for the three-intensity decoy-state MDI-QKD protocol

**Figure 2:**
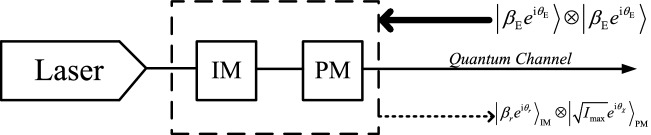
Example of a THA against the IM and the PM of Alice (Bob). For simplicity, we assume that Eve sends Alice (Bob) two high intensity single-mode coherent pulses, each of which is denoted by $\left| {\beta _{\text{E}}{e^{i\theta _{\text{E}}}}} \right\rangle$. One of them targets the IM and the other one targets the PM. We further assume also for simplicity that the back-reflected light from the IM and the PM to Eve is in a product state of two coherent states. One comes from the IM, which we denote by $\left| {{\beta _{r}}{e^{i{\theta _{r}}}}} \right\rangle$, and the other comes from the PM, which has the form $\left| {{\sqrt{I_{\text{max}}} }{e^{i{\theta _{\chi }}}}} \right\rangle$, where *r* and $\chi$ refer to the intensity setting and basis choice, respectively, with $r \in \{\text{s,v,w}\}$ and $\chi \in \{\text{Z,X}\}$. Eve can learn partial information about the intensity settings and the basis choices by separately measuring the states $\left| {{\beta _{r}}{e^{i{\theta _{r}}}}} \right\rangle$ and $\left| {{\sqrt{I_{\text{max}}} }{e^{i{\theta _{\chi }}}}} \right\rangle$.

In the simulation, only for illustration purposes, we assume a particular example of THA, which is shown in Fig. 2. Eve sends Alice (Bob) two high intensity single-mode coherent pulses, each of which is denoted by $\left| {\beta _{\text{E}}{e^{\text{i}\theta _{\text{E}}}}} \right\rangle$, with $\beta _{\text{E}}$ representing the amplitude and $\theta _{\text{E}}$ the phase of the coherent state. One of them targets the IM and the other one targets the PM. For simplicity, we shall also assume that the back-reflected light from both the IM and the PM to Eve is still a coherent state. In so doing, as we show in the Supplementary Information 1, we can obtain simply analytical expressions for those quantities where we apply the trace distance argument. Moreover, we further assume that the back-reflected light from the IM has the form $\left| {{\beta _{r}}{e^{\text{i}{\theta _{r}}}}} \right\rangle$, where the values of the parameters $\beta _{r}$ and $\theta _{r}$ depend on Alice’s and Bob’s intensity settings each given time with $r\in \{\text{s,v,w}\}$, and the back-reflected light from the PM is given by $\left| {{\sqrt{I_{\text{max}}} }{e^{i{\theta _{\chi }}}}} \right\rangle$, where $I_{\text{max}}$ is the maximum intensity of the back-reflected light and $\chi \in \{\text{Z, X}\}$ refers to the basis choice. Note that, here, for simplicity, and in order to compare our simulation results to those in^25^, we assume that Eve’s back-reflected light from the PM only contains the basis information, as already mentioned above. That is, we assume that $|{\Psi _{0,\text{Z}}^i}\rangle _{\text{A',E}}=|{\Psi _{0,\text{Z}}^i}\rangle _\text{A'}\otimes |{\phi _{\text{Z}}}\rangle _{\text{E}}$ and $|{\Psi _{1,\text{Z}}^i}\rangle _{\text{A',E}}=|{\Psi _{1,\text{Z}}^i}\rangle _{\text{A}'}\otimes |{\phi _{\text{Z}}}\rangle _{\text{E}}$, where the state $|{\phi _{\text{Z}}}{\rangle _{\text{E}}}{\text{= }}|\sqrt{{I_{{\text{max}}}}} {e^{i{\theta _{\text{Z}}}}}\rangle$ of Eve’s back-reflected light is the same for both bit values (and similarly for the X basis). Here, the state $|{\Psi _{0,\text{Z}}^i}\rangle _{\text{A',E}}$ ($|{\Psi _{1,\text{Z}}^i}\rangle _{\text{A',E}}$) denotes the joint state of Alice and Eve when Alice uses the Z basis to encode the bit value 0 (1) in the *i*th round of the protocol and the state $|{\Psi _{0,\text{Z}}^i}\rangle _{\text{A}'}$ ($|{\Psi _{1,\text{Z}}^i}\rangle _{\text{A}'}$) denotes the state of Alice in such scenario. Likewise, we assume a similar situation at Bob’s side. Further details can be found in the Supplementary Information 1. To learn partial information about the intensity settings, Eve can measure the state $\left| {{\beta _{r}}{e^{i{\theta _{r}}}}} \right\rangle$, and to learn partial information about the basis choices, Eve can measure the state $\left| {{\sqrt{I_{\text{max}}} }{e^{i{\theta _{\chi }}}}} \right\rangle$. We emphasize, however, that this is just a particular model of a THA that we use it as an example to evaluate the secret key rate in a simple way. It is important to emphasize, however, that our security analysis can be applied to any THA. It remains a very important open question to determine the optimal state that Eve can send to Alice and Bob, as well as to experimentally characterize the identity of the back-reflected light. These questions are generally setup dependent and are beyond the scope of this paper.

In the presence of information leakage, the actual secret key length, $\ell '$, is bounded by

2 $$\begin{aligned} \ell ' \ge \mathop {\max }\limits _{{\Gamma _{\text{AB}}}} \mathop {\min }\limits _{{\Gamma _{\text{E}}}} \ell , \end{aligned}$$

where $\ell$ is given by Eq. (1). Here, $\Gamma _{\text{AB}}$ and $\Gamma _{\text{E}}$ denote the spaces of the parameters controlled by Alice and Bob, and by Eve, respectively. In the simulation, we assume a practically reasonable value for the weakest decoy state, $\gamma ^{\text{w}}=5\times 10^{-4}$, and, without loss of generality, we assume that $\theta _{\text{s}}=0$. The experimental parameters used in the simulations are listed in Table 1. Below we present the simulation results of the secret key rates in three practical cases within the framework of the THA described above. Each case corresponds to a particular model for the back-reflected light.

#### Case 1

In the framework of the THA considered, it is clear that the higher the intensity of the back-reflected light is, the more information Eve can extract. In this first example, we evaluate a worst-case scenario, where Alice and Bob may overestimate the intensity of the back-reflected light leaked to Eve. In particular, we suppose that the intensity ${{\beta _{r}}} ^2$ is always upper bounded by a certain value $I_{\text{max}}$ for all *r* and we conservatively assume that

3 $$\begin{aligned} { {{\beta _{\text{s}}}} ^2} = { {{\beta _{\text{v}}}} ^2} = { {{\beta _{\text{w}}}}^2} ={I_{\text{max }}}. \end{aligned}$$

**Figure 3:**
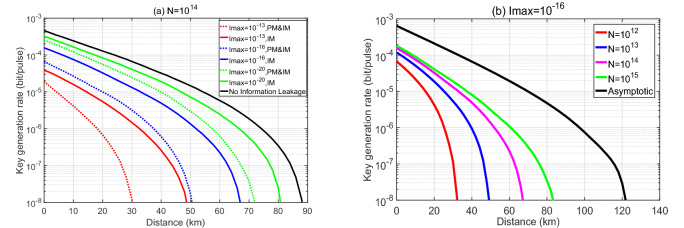
The secret key rate in logarithmic scale as a function of the distance in Case 1 for the three-intensity protocol. (**a**) Here we consider a fixed value of the total number of transmitted pulses, $N=10^{14}$ and various values for the intensity $I_\text{max}$. (**b**) Here we fix $I_\text{max}=10^{-16}$ and consider various values for *N*. Moreover, we evaluate information leakage from the IM only.

The simulation result of the secret key rate, $\ell '/N$, as a function of the transmission distance between Alice and Bob in this case is shown in Fig. 3a for a fixed value of the total number of transmitted pulses, $N=10^{14}$. In this figure, the black solid line represents the key rate in the situation where there is no information leakage, namely $I_{\text{max}}=0$, and the different colored lines correspond to different amounts of information leakage. More precisely, the colored solid lines represent the key rates in the presence of a THA against only the IM. If we compare these results to the longest achievable distance without information leakage, which is about 88 km, we find that now the secret key rate vanishes at about 48 km even when $I_{\text{max}}$ is as small as $10^{-13}$. The colored dotted lines represent the secret key rates in the presence of a THA against both the IM and the PM. Now the secret key rates are obviously lower than the ones corresponding to a THA against only the IM. For example, when $I_{\text{max}}=10^{-13}$ the secret key rate now vanishes at only 30 km. These results highlight the strong effect that information leakage (even when is very tiny) can have on the performance of MDI-QKD.

As already observed in the finite-key analysis for decoy-state QKD^25^, here we also find that in MDI-QKD Alice and Bob need to discard part of their data (on average about $Np_{\text{X}_{\text{A}_{\text{c}}}}$ pulse pairs) to estimate the phase error rate when there is information leakage from the PM. In our simulation, we find that the optimal value of $p_{\text{Z}_{{\text{A}}_{\text{c}}}}$ typically lies in the interval $\left[ 0.65,0.9 \right]$. Note that, compared to the simulation result in^25^, we have that the value of $p_{\text{Z}_{\text{A}_{\text{c}}}}$ is typically smaller in the MDI-QKD protocol, which means that MDI-QKD has to sacrifice a bigger proportion of data than in the case of the standard decoy-state QKD protocol to estimate the phase error rate.

Also, we find that MDI-QKD seems to be more sensitive to information leakage. In order to obtain a certain performance, the value of $I_{\text{max}}$ in MDI-QKD is roughly the square of that in standard decoy-state QKD due to the fact that in MDI-QKD there are two leaky sources (Alice and Bob) instead of only one leaky source. Thus, to implement the MDI-QKD protocol, both Alice and Bob need to carefully isolate their devices from the external environment to guarantee the security of the system.

In Fig. 3b, the different colored lines show the secret key rate as a function of the distance for a fixed value $I_{\text{max}}=10^{-16}$ and for different total numbers of transmitted pulses. Here, for simplicity, we only plot the key rates against information leakage from the IM and omit the results when there is also information leakage from the PM as they are similar to those shown in Fig. 3b. That is, in this figure we can see the effect of the information leakage as a function of the number of transmitted pulses. For example, when $I_{\text{max}}=10^{-16}$, the longest achievable distance is about 84 km when the total number of transmitted pulses is $N=10^{15}$. However, when $N=10^{12}$, this distance decreases to 32 km. Our results indicate that the finite-key effect has a much bigger impact on the secret key rate in the presence of information leakage^27^. The reason for this is mainly that, in order to estimate the statistical fluctuations for a finite sampling size in the presence of information leakage from the IM, our methodology relies on applying Azuma’s inequality^34^ to the total number of *transmitted pulses*. In contrast, when there is no information leakage from the IM, one can apply Azuma’s inequality to the number of *pulses detected*. This is so because in this latter case, one can assume a counterfactual scenario where Alice and Bob select their intensity settings a posteriori, i.e., after the relay has detected the successful events. As a consequence, the performance of MDI-QKD in the finite-key regime is comparatively worse in the presence of information leakage from the IM. Note that for the case of information leakage from the PM, we actually apply Azuma’s inequality to the number of the detected events.

**Figure 4:**
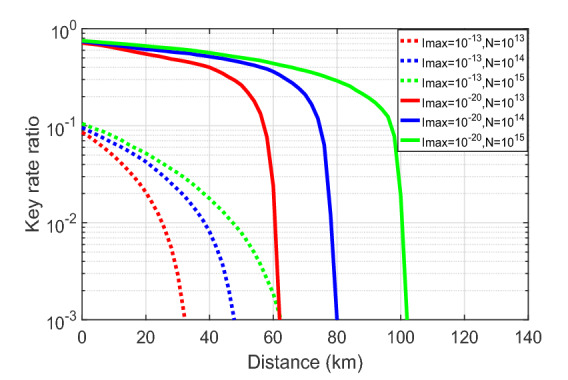
The ratio ($\ell '_{I_{\text{max}}>0}/\ell '_{I_{\text{max}}=0}$) between the secret key rates in logarithmic scale with and without information leakage as a function of the distance for two fixed positive values of $I_{\text{max}}=\{10^{-13},~10^{-20}\}$ and various values for *N*.

To further illustrate how the information leakage affects the secret key rate as a function of the number of transmitted pulses, in Fig. 4 we plot the ratio ($\ell '_{I_{\text{max}}>0}/\ell '_{I_{\text{max}}=0}$) between the secret key rates for two fixed positive values of information leakage, $I_{\text{max}}=\{10^{-13},~10^{-20}\}$ and those when $I_{\text{max}}=0$ (i.e., when there is no information leakage) for different values of *N*. Here, for simplicity, we disregard again the information leakage from the PM. From Fig. 4 one can see that given a fixed distance and a fixed value of *N*, the ratio when $I_{\text{max}}=10^{-13}$ is at least one order of magnitude lower than that when $I_{\text{max}}=10^{-20}$. And the ratio when $I_\text{max}=10^{-13}$ drops faster as the distance increases than that when $I_{\text{max}}=10^{-20}$. For instance, if we focus on the red lines, from 0 to 30 km, the ratio drops from about $10^{-1}$ to $10^{-3}$ when $I_{\text{max}}=10^{-13}$ (i.e., two orders of magnitude) while the ratio drops only from 0.71 to 0.49 (i.e., of the same order of magnitude) when $I_{\text{max}}=10^{-20}$. This suggests that the effect of information leakage increases when *N* decreases, and the finite-size effect is amplified when the amount of information leakage increases. We remark that the simulation results for the other two cases that we consider next are analogous to those of Fig. 4 and thus we omit them in the next two subsections.

#### Case 2

In the previous case, we considered a conservative scenario for Alice and Bob, where the intensity of the back-reflected light is maximal and independent of the settings of the IM. Thus, the amount of information leaked might be overestimated, which results in a relatively pessimistic lower bound on the secret key rate. However, in practice, the input light of Eve may also go through the IM. As a consequence, the back-reflected light could be modulated in the same manner as the senders’ pulses during the state preparation process. In this case, we have that

4 $$ \beta _{{\text{s}}} ^{2}  = \frac{{\gamma ^{{\text{s}}} }}{{\gamma ^{{\text{v}}} }}\beta _{{\text{v}}} ^{2}  = \frac{{\gamma ^{{\text{s}}} }}{{\gamma ^{{\text{w}}} }}\beta _{{\text{w}}} ^{2}  = I_{{\max }} . $$

That is, here we assume that the maximum amount of information leakage comes from the largest intensity setting of the senders, namely ${I_{\max }} = { {{\beta _{\text{s}}}} ^2}$. The intensity of the back-reflected light corresponding to the other intensity settings fulfills the conditions: ${{\beta _{\text{s}}}} ^2/{{\beta _{\text{v}}}} ^2={\gamma ^{\text{s}}}/{\gamma ^{\text{v}}}$ and ${{\beta _{\text{s}}}} ^2/{{\beta _{\text{w}}}} ^2={\gamma ^{\text{s}}}/{\gamma ^{\text{w}}}$.

The simulation results of the secret key rate are shown in Fig. 1 in the Supplementary Information. The behavior of the curves is very similar to those in Case 1, and in the simulation we find that the optimized value of $p_{\text{Z}_{\text{A}_{\text{c}}}}$ is similar as well. One main difference is that with the same experimental parameters the secret key rate is now a little bit higher and can go a bit further than that in Case 1. For example, when the total number of transmitted pulses is $10^{14}$ and the maximum intensity of the back-reflected light is $I_{\text{max}}=10^{-13}$, we find that the secret key is positive up to about 54 km while in Case 1 this distance is 48 km in the presence of information leakage only from the IM.

#### Case 3

In this case we consider a more favorable situation for Alice and Bob where they implement an additional step to randomize the phase of each signal going out of their transmitters including the back-reflected light to Eve. Moreover, we optimistically assume that there is no information leakage from this phase randomization step. Furthermore, we suppose that the amplitudes $\beta _{k}$ still satisfy Eq. (4) like in the previous case. Then, we have that the state of Eve’s back-reflected light from the IM and the PM are given by:

5 $$\begin{aligned} \begin{array}{*{20}{l}} {\rho _{{\gamma ^k}}} = {e^{ -(\beta _{k})^2}}\sum \limits _{n = 0}^\infty {\frac{{(\beta _{k})^2}}{{n!}}} |n\rangle \langle n|,\\ {{\rho _{{I_{\max }}}} = {e^{ - {I_{\max }}}}\sum \limits _{n = 0}^\infty {\frac{{{I_{\max }}}}{{n!}}} |n\rangle \langle n|, }\end{array} \end{aligned}$$

respectively.

This means that the information about Alice’s and Bob’s inner settings can only be leaked via the amplitudes of the back-reflected light but Eve cannot obtain any information from its phase. We remark, however, that here we consider a model which is slightly different from the ones considered in previous works^24,25^. To be precise, in Refs.^24,25^ the authors consider that the phase randomization step is only applied to the back-reflected light from the IM. However, here we consider that this step is applied to the back-reflected light from both the IM and the PM. This means that, now Eve cannot exploit any information leakage from the PM, but only information leakage from the IM as the state $\rho _{I_{\text{max}}}$ does not depend on the basis choice.

The simulation results of the secret key rate are shown in Fig. 2 in the Supplementary Information. Here, we find that the typical interval where $p_{\text{Z}_{{\text{A}}_{\text{c}}}}$ lies is $\left[ 0.71,0.93 \right]$. Compared to the secret key rate shown in the previous two cases, now the secret key rate is obviously improved. For example, when the total number of transmitted pulses is $N=10^{14}$ and $I_{\text{max}}=10^{-7}$, the secret key rate remains positive up to about 62 km. In comparison, the maximum achievable distance with the same number of transmitted pulses and assuming an $I_{\text{max}}$ as low as $10^{-13}$ is only about 36 km in Case 2, and it is even worse in Case 1.

In practice, however, Eve might also perform a THA against the phase randomization step to obtain some information about the random phase applied by Alice and Bob each given time. This will obviously reduce the benefit of the phase randomization step. One could also analyze this last scenario with the techniques in this paper, but for simplicity we omit it here.

### Simulation results for the four-intensity decoy-state MDI-QKD protocol

**Figure 5:**
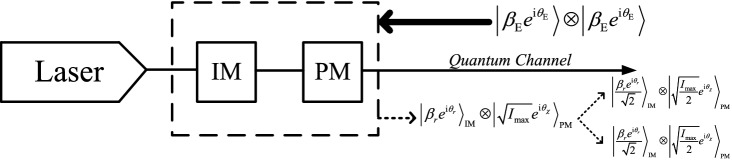
Example of a THA against correlated IM and PM of Alice (Bob). The simulation is similar to that in Fig. 2 but now $r \in \{\text{s,v,w,0}\}$. Moreover, we assume that Eve splits the joint back-reflected light into two parts by means of a 50:50 beamsplitter, one part is to learn information about the intensity settings and the other part is used to learn information about the basis choices.

In what follows, for illustration purposes we consider a particular example of the THA considered in the previous section, which is shown in Fig. 5. Now, however, the back-reflected light from the IM has the form $\left| {{\beta _{r}}{e^{i{\theta _{r}}}}} \right\rangle$ with $r \in \{\text{s,v,w,0}\}$. Moreover, since the IM and the PM are correlated, Eve can jointly measure the states $\left| {{\beta _{r}}{e^{i{\theta _{r}}}}} \right\rangle$ and $\left| {{\sqrt{I_{\text{max}}} }{e^{i{\theta _{\chi }}}}} \right\rangle$, which is the back-reflected light from the PM with $\chi \in \{\text{Z,X}\}$, to extract partial information about both the intensity settings and the basis choices. Particularly, we shall consider that Eve splits the joint back-reflected light $\left| {{\beta _{r}}{e^{i{\theta _{r}}}}} \right\rangle \otimes \left| {{\sqrt{I_{\text{max}}} }{e^{i{\theta _{\chi }}}}} \right\rangle$ into two parts by means of a 50:50 beamsplitter, one part is used to learn partial information about the intensity settings and the other part is used to learn partial information about the basis choices. We remark, however, that our method to estimate the phase error rate could be applied to any strategy applied by Eve. Importantly, to have a fair comparison with the simulation results shown in the previous section, we assume that the amount of information leaked to Eve in both protocols is the same. That is, we assume that the intensity of the back-reflected light is equal in both cases.

Note that since the information leakage from the IM and the PM is correlated, in the following figures, we plot the secret key rates in the presence of information leakage from both devices.

#### Case 1

**Figure 6:**
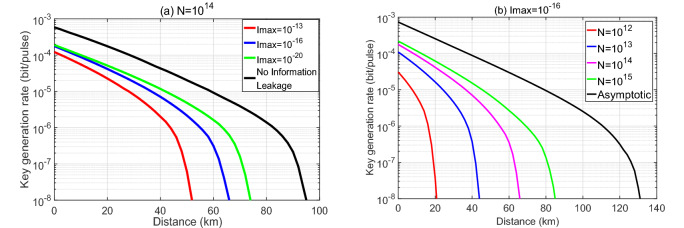
The secret key rate in logarithmic scale as a function of the distance in Case 1 for the four-intensity protocol. (**a**) Here we consider a fixed value of the total number of transmitted pulses, $N=10^{14}$ and various values for the intensity $I_\text{max}$. (**b**) Here we fix $I_{\text{max}}=10^{-16}$ and consider various values for *N*.

The simulation result of the secret key rate, $\ell '/N$, as a function of the transmission distance between Alice and Bob in this case is shown in Fig. 6a for a fixed value of the total number of transmitted pulses, $N=10^{14}$. The black solid line represents the key rate in the situation where there is no information leakage, and the different colored lines correspond to different amounts of information leakage. The longest achievable distance without information leakage is about 96 km. When $I_{\text{max}}=10^{-13}$, the secret key rate vanishes at about 52 km. In the simulation, we find that in this case the optimized value of $p_{\text{Z}_{\text{A}_{\text{c}}}}$ typically lies in the interval $\left[ 0.75,0.94 \right]$. That is, in this protocol Alice and Bob can sacrifice a smaller proportion of the data than that in the symmetric three-intensity decoy-state MDI-QKD protocol (where, as we have shown in the previous section, the typical interval of the optimized value of $p_{\text{Z}_{\text{A}_{\text{c}}}}$ is $\left[ 0.65,0.9 \right]$).

Figure 6b shows the secret key rates as a function of the distance for a fixed value $I_{\text{max}}=10^{-16}$ for different total numbers of transmitted pulses. For example, the longest achievable distance is about 84 km when the total number of transmitted pulses is $N=10^{15}$. However, when $N=10^{12}$, this distance decreases to 21 km.

**Figure 7:**
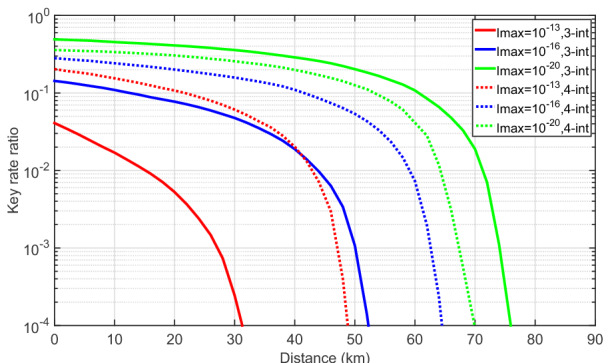
The ratio ($\ell '_{I_{\text{max}}>0}/\ell '_{I_{\text{max}}=0}$) between the secret key rates in logarithmic scale with and without information leakage as a function of the distance for a fixed number, $N=10^{14}$, of transmitted pulses in the two protocols (3-int and 4-int represent the three-intensity decoy state MDI-QKD protocol and the four-intensity decoy-state MDI protocol that we consider, respectively).

To further compare the effect of the information leakage on the secret key rate in the two MDI-QKD protocols that we consider, we plot the ratio ($\ell '_{I_{\text{max}}>0}/\ell '_{I_{\text{max}}=0}$) between the secret key rates for different positive values of information leakage, $I_{\text{max}}$, and the secret key rate when there is no information leakage, i.e., $I_{\text{max}}=0$, given a fixed total number of transmitted pulses, say, $N=10^{14}$ in Fig. 7. The solid and dotted lines represent the ratios in the symmetric three-intensity decoy-state MDI-QKD protocol and in the four-intensity decoy-state MDI-QKD protocol, respectively. In the following, for simplicity, let us denote these two protocols by ‘3-int’ and ‘4-int’, respectively. The result in Fig. 7 indicates that when the amount of information leakage is small enough, for instance, $I_{\text{max}}=10^{-20}$, the impact of the information leakage on the 3-int protocol is smaller than that on the 4-int protocol as the green solid line is always above the green dotted line. However, the key rate ratio drops much faster as the amount of information leakage increases in the 3-int protocol than that in the 4-int protocol. From Fig. 7, we find that when $I_{\text{max}}=10^{-16}$ and $I_{\text{max}}=10^{-13}$, the ratio in the 4-int protocol is bigger than that in the 3-int protocol. That is, when $I_{\text{max}}$ increases, the effect of information leakage becomes more relevant on the 3-int protocol than that on the 4-int protocol given a fixed total number of transmitted pulses.

The intuition for this behaviour could be the following: from Figs. 2 and 5, we can see that the back-reflected light from the PM is the same for the 3-int and 4-int protocol. Now suppose that in the 4-int protocol Eve measures the back-reflected light from the IM and the PM independently instead of splitting the back-reflected light with a 50:50 BS. Then she learns the same information from the PM in both protocols. However, it may be more difficult for Eve to learn the information from the IM in the 4-int protocol than in the 3-int protocol because she needs to distinguish between four states in the former but she only needs to distinguish between three states in the latter. In this case, the 4-int protocol is more robust against information leakage than the 3-int protocol for all values of $I_{\text{max}}$. Nevertheless, if Eve exploits the correlations between the back-reflected light from the IM and the PM, then which protocol is more robust seems to depend on the value of $I_{\text{max}}$. In addition, note that the results illustrated in Fig. 7 consider the case where Eve splits the back-reflected light with a 50:50 BS, which might not be the optimal option for the example of THAs evaluated.

#### Case 2

The simulation results of the secret key rate as a function of the transmission distance are shown in Fig. 3 in the Supplementary Information. The behavior of the curves is very similar to those in case 1, and in the simulation we find that the optimized value of $p_{\text{Z}_{\text{A}_{\text{c}}}}$ is also similar. One main difference is that with the same experimental parameters the secret key rate is a little higher and the achievable distance is a little longer than those in Case 1. For example, when the total number of transmitted pulses is $N=10^{14}$ and the maximum intensity of the back-reflected light is $I_{\text{max}}=10^{-13}$, now we find that the secret key is positive up to about 57 km while in Case 1 this distance is 52 km.

Here we omit the comparison of the key rate ratios between the two protocols as the result in this case is similar to that shown in Fig. 7. And for the same reason, we omit such comparison in Case 3 as well.

#### Case 3

The simulation results of the secret key rate as a function of the transmission distance are shown in Fig. 4 in the Supplementary Information. Here, we find that the typical interval that $p_{\text{Z}_{{\text{A}}_{\text{c}}}}$ lies in is $\left[ 0.86,0.99 \right]$. Compared to the secret key rates shown in the previous two cases, now it is obviously improved. For example, when the total number of transmitted pulses is $N=10^{14}$ and $I_{\text{max}}=10^{-7}$, the secret key rate remains positive up to about 66 km. In comparison, the maximal achievable distance with the same number of transmitted pulses and assuming an $I_{\text{max}}$ as low as $10^{-13}$ is only about 57 km (52 km) in Case 2 (Case 1). As discussed previously, this is because the phase randomization step removes the information leaked in the phase of the output states to Eve.

## Conclusion and discussion

In this paper, we have quantitatively analyzed the security of two decoy-state MDI-QKD protocols with leaky sources in the finite-key regime. Specially, we have simulated the secret key rate under three particular examples of THA, where Eve sends coherent pulses of light to probe the intensity modulators and phase modulators of the legitimate parties. Similar to the analysis presented in^25^, we have introduced an additional post-processing step in the actual protocol where Alice and Bob sacrifice part of their data. This step is necessary for the security proof to go through. Our simulation results suggest that MDI-QKD is more sensitive to information leakage than standard decoy-state QKD, but is possible to distill secure keys from leaky sources within a reasonable time frame of signal transmission given that Alice’s and Bob’s sources are sufficiently isolated. Furthermore, we have found that when the amount of information leakage is small enough, the effect of information leakage has a bigger impact on the four-intensity decoy-state MDI-QKD protocol than on the symmetric three-intensity decoy-state MDI-QKD protocol. However, when the amount of information leakage increases, the four-intensity MDI-QKD protocol becomes more robust against information leakage than the symmetric three-intensity MDI-QKD protocol.

We note that Ref.^41^ introduced a security analysis for MDI-QKD which does not have to characterize the states emitted by only assuming that the generated signals live in a qubit space. While this analysis might certainly have some advantages in some scenarios (e.g., when evaluating state preparation flaws), it cannot be applied to the situation we study here with leaky sources. Indeed, due to the presence of side-channels, the emitted signals are not qubits but higher dimensional signals. This means that, in its current formulation the work in Ref.^41^ does not apply to the scenario that we evaluate and cannot take information leakage into consideration.

We emphasize that the methods introduced in this paper are completely general and can be applied to any information leakage, not necessarily in the form of coherent states. We have assumed this particular model only for simplicity in order to perform simulations.

In this context it would be interesting to consider a stronger THA, where Eve sends entangled probe states to Alice’s and Bob’s sources instead of sending them independent bright pulses. In such a scenario, by performing a joint measurement on the outgoing states as well as on her ancilla system, Eve might be able to extract more information about Alice’s and Bob’s internal settings than what has been presented in this paper. This case, however, is beyond the scope of this work but could be evaluated with the techniques that have been introduced in this paper.

**Table 1:** Experimental parameters used in the simulations.

$e_{\text{d}}$	$p_{\text{d}}$	$\eta _{\text{det}}$	$\alpha$	$f_{\text{EC}}$
$1\%$	$5\times 10^{-6}$	0.25	0.2	1.2

The parameter $e_{\text{d}}$ is the intrinsic error rate due to the misalignment of the MDI-QKD system; $p_{\text{d}}$ is the dark count rate of the relay’s detectors, which we assume is equal for all of them; $\eta _{\text{det}}$ is the overall detection efficiency of the relay’s receiver; $\alpha$ is the loss coefficient of the channel measured in dB/km; and $f_{\text{EC}}$ is the efficiency of the error correction code.

## Supplementary Information

Supplementary Information.
