# Supplementary material for: Measurement-device-independent quantum key distribution with leaky sources
Source: Sci Rep. 2021 Jan 18;11:1678. doi: 10.1038/s41598-021-81003-2 (PMC7813839; doi:10.1038/s41598-021-81003-2)
Supplement: Supplementary file 1 — Supplementary Information. [file 41598_2021_81003_MOESM1_ESM.pdf]

Supplementary Information for Measurement-Device-Independent Quantum Key Distribution with Leaky Sources

Weilong Wang[1,2,3*], Kiyoshi Tamaki[4], and Marcos Curty[1]

[1]EI Telecomunicación, Department of Signal Theory and Communications, University of Vigo, Vigo E-36310, Spain
[2]State Key Laboratory of Mathematical Engineering and Advanced Computing, Zhengzhou, Henan, 450001, China
[3]Henan Key Laboratory of Network Cryptography Technology, Zhengzhou, Henan, 450001, China
[4]Faculty of Engineering, University of Toyama, Gofuku 3190, Toyama 930-8555, Japan
[*]wwang@com.uvigo.es

ABSTRACT

In this Supplementary Information, we present the assumptions we make on the protocol together with the detailed parameter estimation methods and calculations for the three-intensity and four-intensity decoy-state MDI-QKD protocols.

Assumptions on the protocol

Below we introduce the assumptions we make on the users' devices in the absence of information leakage:

1. Each of Alice and Bob generates perfect phase-randomized weak coherent pulses (WCPs) of the form

$$\rho^{\gamma^j} = \sum_{n=0}^{\infty} p_n^j |n\rangle\langle n|, \tag{1}$$

where $p_n^j = \left(\gamma^j\right)^n e^{-\gamma^j}/n!$ is the probability that Alice (Bob) sends an n-photon pulse given that she (he) selects the intensity setting γ^j, and $|n\rangle$ denotes a Fock state with n photons.

2. The state of a pulse generated by Alice (Bob) is in a single mode and the joint state of all the pulses generated by Alice (Bob) is in a tensor product. That is, we assume that the pulses are not correlated with each other when there is no information leakage. We note that the scenario where the users generate multi-mode signals could be evaluated with the techniques recently introduced in[1], while the case of correlated pulses could be analyzed with the techniques developed in[2].

3. There are no intensity fluctuations, *i.e.*, the intensity of the pulses generated by Alice and Bob is precisely γ^j.

4. Alice and Bob can perfectly encode the bit and basis information, *i.e.*, there are no state preparation flaws. We remark that the case of state preparation flaws could be studied using the methods presented in[1,3]

5. Alice's and Bob's phase modulators modulate only the phase of the pulses, and their intensity modulators modulate only the intensity of the pulses. That is, the information is only encoded in the desired degrees of freedom of the signals.

Parameter estimation method for the three-intensity protocol with leaky sources

THA against the intensity modulator

Here, we provide the detailed calculations to analyze a THA targeted against the intensity modulator (IM), which is used to generate decoy states. As already explained in the main text, to simplify the analysis, we first consider an asymptotic scenario where Alice and Bob send an infinite number of pulses.

Table 1. Quantum systems defined in a THA against the IM

$E_{\rm p}^{\rm A}$ ($E_{\rm p}^{\rm B}$)	Eve's probe system sent to Alice (Bob)
$E_{\rm a}^{\rm A}$ ($E_{\rm a}^{\rm B}$)	Eve's ancillary system which could be entangled with $E_{\rm p}^{\rm A}$ ($E_{\rm p}^{\rm B}$)
$E_{\rm p}^{\rm A'}$ ($E_{\rm p}^{\rm B'}$)	Back-reflected light from $E_{\rm p}^{\rm A}$ ($E_{\rm p}^{\rm B}$)
$\rho_{\chi,nm}^{\gamma^{j_{\rm A}}\gamma^{j_{\rm B}},i}$	Normalized joint state of Alice's n-photon pulse and Bob's m-photon pulse when they select the intensity settings $\gamma^{j_{\rm A}}$ and $\gamma^{j_{\rm B}}$, respectively, with the same basis choice χ, and the systems $E_{\rm a}^{\rm A}$, $E_{\rm a}^{\rm B}$, $E_{\rm p}^{\rm A'}$, $E_{\rm p}^{\rm B'}$ in the ith trial

The Asymptotic Limit

Let us denote the intensity settings of Alice and Bob in the ith trial of the protocol by $\gamma^{j_{\rm A},i}$ and $\gamma^{j_{\rm B},i}$ with $j_{\rm A}, j_{\rm B} \in \{{\rm s,\ v,\ w}\}$. Also, suppose that Eve prepares and sends Alice (Bob) a probe system $E_{\rm p}^{\rm A}$ ($E_{\rm p}^{\rm B}$) which could be entangled with an ancilla system $E_{\rm a}^{\rm A}$ ($E_{\rm a}^{\rm B}$) stored in her quantum memory. Here, for simplicity, we shall assume that the state of systems $E_{\rm p}^{\rm A}$ and $E_{\rm a}^{\rm A}$ is not correlated with that of systems $E_{\rm p}^{\rm B}$ and $E_{\rm a}^{\rm B}$. However, we remark that our formalism can be adapted to the correlated case as well. Afterwards, Eve performs a joint measurement on the pulses emitted by Alice (Bob) together with the system $E_{\rm a}^{\rm A}$ ($E_{\rm a}^{\rm B}$) and the back-reflected light from $E_{\rm p}^{\rm A}$ ($E_{\rm p}^{\rm B}$), which is denoted by $E_{\rm p}^{\rm A'}$ ($E_{\rm p}^{\rm B'}$). Let $\rho_{\chi,nm}^{\gamma^{j_{\rm A}}\gamma^{j_{\rm B}},i}$ represent the normalized joint state of Alice's n-photon pulse and Bob's m-photon pulse when they select the intensity settings $\gamma^{j_{\rm A}}$ and $\gamma^{j_{\rm B}}$, respectively, with the same basis choice χ, together with the systems $E_{\rm a}^{\rm A}$, $E_{\rm a}^{\rm B}$, $E_{\rm p}^{\rm A'}$ and $E_{\rm p}^{\rm B'}$ in the ith trial. Now, it is important to determine how well Eve can distinguish the states $\rho_{\chi,nm}^{\gamma^{j_{\rm A}}\gamma^{j_{\rm B}},i}$ for different intensity settings. In particular, we consider how well she can distinguish the intensity settings $\gamma^{j_{\rm A}}$ and $\gamma^{j_{\rm B}}$ from, say, $\gamma^{k_{\rm A}}$ and $\gamma^{k_{\rm B}}$ or $\gamma^{l_{\rm A}}$ and $\gamma^{l_{\rm B}}$ in each trial, where $j_{\rm A}, j_{\rm B}, k_{\rm A}, k_{\rm B}, l_{\rm A}, l_{\rm B} \in \{{\rm s,v,w}\}$ and $j_{\rm A} \neq k_{\rm A}, l_{\rm A}$ and $j_{\rm B} \neq k_{\rm B}, l_{\rm B}$. All these quantum systems are listed in Table. 1.

According to the trace distance argument[4–6], we have that

$$\sum_{\omega\in\Omega}\left|P(\omega|\rho_{\chi,nm}^{\gamma^{j_{\rm A}}\gamma^{j_{\rm B}},i}) - P(\omega|\sigma_{\chi,nm}^{\gamma^{k_{\rm A}l_{\rm A}}\gamma^{k_{\rm B}l_{\rm B}},i})\right| \le D_{\chi,nm}^{j_{\rm A}j_{\rm B},k_{\rm A}k_{\rm B}l_{\rm A}l_{\rm B},i}, \tag{2}$$

where Ω is a set of physical events that satisfies $\sum_{\omega\in\Omega} P(\omega) = 1$, $P(\omega|\rho)$ is the conditional probability that the event ω occurs given a state ρ, and $\sigma_{\chi,nm}^{\gamma^{k_{\rm A}l_{\rm A}}\gamma^{k_{\rm B}l_{\rm B}},i} := q_{nmkl}\rho_{\chi,nm}^{\gamma^{k_{\rm A}}\gamma^{k_{\rm B}},i} + (1-q_{nmkl})\rho_{\chi,nm}^{\gamma^{l_{\rm A}}\gamma^{l_{\rm B}},i}$, where $q_{nmkl} = p_{k_{\rm A}}p_{k_{\rm B}}p_n^{k_{\rm A}}p_m^{k_{\rm B}}/(p_{k_{\rm A}}p_{k_{\rm B}}p_n^{k_{\rm A}}p_m^{k_{\rm B}} + p_{l_{\rm A}}p_{l_{\rm B}}p_n^{l_{\rm A}}p_m^{l_{\rm B}})$ is a normalization factor. That is, $\sigma_{\chi,nm}^{\gamma^{k_{\rm A}l_{\rm A}}\gamma^{k_{\rm B}l_{\rm B}},i}$ corresponds to the normalized joint state of Alice's n-photon pulse and Bob's m-photon pulse when they select the intensity settings $\gamma^{k_{\rm A}}$ and $\gamma^{k_{\rm B}}$ or $\gamma^{l_{\rm A}}$ and $\gamma^{l_{\rm B}}$, respectively, with the same basis choice χ, together with the systems $E_{\rm a}^{\rm A}$, $E_{\rm a}^{\rm B}$, $E_{\rm p}^{\rm A'}$ and $E_{\rm p}^{\rm B'}$ in the ith trial. We remark that more general cases with up to eight different combinations of intensity settings (*i.e.*, all combinations of intensity settings except $\gamma^{j_{\rm A}}$ and $\gamma^{j_{\rm B}}$) could be considered here. The parameter $D_{\chi,nm}^{j_{\rm A}j_{\rm B},k_{\rm A}k_{\rm B}l_{\rm A}l_{\rm B},i}$ denotes the trace distance between the states $\rho_{\chi,nm}^{\gamma^{j_{\rm A}}\gamma^{j_{\rm B}},i}$ and $\sigma_{\chi,nm}^{\gamma^{k_{\rm A}l_{\rm A}}\gamma^{k_{\rm B}l_{\rm B}},i}$ and it is given by

$$D_{\chi,nm}^{j_{\rm A}j_{\rm B},k_{\rm A}k_{\rm B}l_{\rm A}l_{\rm B},i} = \frac{1}{2}{\rm Tr}\left[\sqrt{(\rho_{\chi,nm}^{\gamma^{j_{\rm A}}\gamma^{j_{\rm B}},i} - \sigma_{\chi,nm}^{\gamma^{k_{\rm A}l_{\rm A}}\gamma^{k_{\rm B}l_{\rm B}},i})^2}\right]. \tag{3}$$

Basically, Eq. (2) quantifies how well the states $\rho_{\chi,nm}^{\gamma^{j_{\rm A}}\gamma^{j_{\rm B}},i}$ and $\sigma_{\chi,nm}^{\gamma^{k_{\rm A}l_{\rm A}}\gamma^{k_{\rm B}l_{\rm B}},i}$ can be distinguished from each other.

Specially, let $\Omega = \{{\rm click,\ no\ click}\}$, where "click" ("no click") represents a successful (unsuccessful) measurement event at the relay and let ${\rm Pr}^i({\rm click}|nm, j_{\rm A}j_{\rm B}, \chi)$ denote the conditional probability that the relay obtains a "click" given the state $\rho_{\chi,nm}^{\gamma^{j_{\rm A}}\gamma^{j_{\rm B}},i}$. Then according to Eq. (2) we have that

$$\begin{aligned}&\left|{\rm Pr}^i({\rm click}|nm, j_{\rm A}j_{\rm B}, \chi) - \left[q_{nmkl}{\rm Pr}^i({\rm click}|nm, k_{\rm A}k_{\rm B}, \chi) + (1-q_{nmkl}){\rm Pr}^i({\rm click}|nm, l_{\rm A}l_{\rm B}, \chi)\right]\right| \\ &\le D_{\chi,nm}^{j_{\rm A}j_{\rm B},k_{\rm A}k_{\rm B}l_{\rm A}l_{\rm B},i}.\end{aligned} \tag{4}$$

By multiplying both sides of Eq. (4) by $p_{j_{\rm A}}p_{j_{\rm B}}p_n^{j_{\rm A}}p_m^{j_{\rm B}}$ and taking the sum over $i = \{1,2,...,N_\chi\}$, where N_χ denotes the number of events when both Alice and Bob choose the χ basis, we obtain

$$\begin{aligned}&\left|\sum_{i=1}^{N_\chi}{\rm Pr}^i({\rm click}, nm, j_{\rm A}j_{\rm B}|\chi) - p_{j_{\rm A}}p_{j_{\rm B}}p_n^{j_{\rm A}}p_m^{j_{\rm B}}\sum_{i=1}^{N_\chi}\left[q_{nmkl}\frac{{\rm Pr}^i({\rm click},nm,k_{\rm A}k_{\rm B}|\chi)}{p_{k_{\rm A}}p_{k_{\rm B}}p_n^{k_{\rm A}}p_n^{k_{\rm B}}} + (1-q_{nmkl})\frac{{\rm Pr}^i({\rm click},nm,l_{\rm A}l_{\rm B}|\chi)}{p_{l_{\rm A}}p_{l_{\rm B}}p_n^{l_{\rm A}}p_m^{l_{\rm B}}}\right]\right| \\ &\le p_{j_{\rm A}}p_{j_{\rm B}}p_n^{j_{\rm A}}p_m^{j_{\rm B}}N_\chi D_{\chi,nm}^{j_{\rm A}j_{\rm B},k_{\rm A}k_{\rm B}l_{\rm A}l_{\rm B}},\end{aligned} \tag{5}$$

where $\Pr^i(\text{click}, nm, j_A j_B | \chi)$ is the conditional probability that Alice selects the intensity setting γ^{j_A} and sends an n-photon pulse, Bob selects the intensity setting γ^{j_B} and sends an m-photon pulse, and the relay obtains a successful measurement result given that they both select the χ basis. In Eq. (5) we have used the definition $D_{\chi,nm}^{j_A j_B,k_A k_B l_A l_B} = \frac{1}{N_\chi}\sum_{i=1}^{N_\chi} D_{\chi,nm}^{j_A j_B,k_A k_B l_A l_B,i}$.

The quantity $\sum_{i=1}^{N_\chi} \Pr^i(\text{click}, nm, j_A j_B | \chi)$ corresponds to the conditional expected number of events, which we shall denote by $\mathscr{E}_{\text{click},nm,j_A j_B|\chi}$. Then, we have that Eq. (5) can be rewritten as

$$\left|\mathscr{E}_{\text{click},nm,j_A j_B|\chi} - \left[q_{nmkl}\frac{p_{j_A}p_{j_B}p_n^{j_A}p_m^{j_B}}{p_{k_A}p_{k_B}p_n^{k_A}p_m^{k_B}}\mathscr{E}_{\text{click},nm,k_A k_B|\chi} + (1-q_{nmkl})\frac{p_{j_A}p_{j_B}p_n^{j_A}p_m^{j_B}}{p_{l_A}p_{l_B}p_n^{l_A}p_m^{l_B}}\mathscr{E}_{\text{click},nm,l_A l_B|\chi}\right]\right| \leq p_{j_A}p_{j_B}p_n^{j_A}p_m^{j_B}N_\chi D_{\chi,nm}^{j_A j_B,k_A k_B l_A l_B}. \tag{6}$$

If we take, for instance, the particular case where $l_A = k_A$ and $l_B = k_B$, then Eq. (6) can be written as:

$$\left|\mathscr{E}_{\text{click},nm,j_A j_B|\chi} - \frac{p_{j_A}p_{j_B}p_n^{j_A}p_m^{j_B}}{p_{k_A}p_{k_B}p_n^{k_A}p_m^{k_B}}\mathscr{E}_{\text{click},nm,k_A k_B|\chi}\right| \leq p_{j_A}p_{j_B}p_n^{j_A}p_m^{j_B}N_\chi D_{\chi,nm}^{j_A j_B,k_A k_B}, \tag{7}$$

where

$$D_{\chi,nm}^{j_A j_B,k_A k_B} = \frac{1}{N_\chi}\sum_{i=1}^{N_\chi} D_{\chi,nm}^{j_A j_B,k_A k_B,i} := \frac{1}{N_\chi}\sum_{i=1}^{N_\chi}\text{Tr}\left[\sqrt{\left(\rho_{\chi,nm}^{\gamma^{j_A}\gamma^{j_B},i} - \rho_{\chi,nm}^{\gamma^{k_A}\gamma^{k_B},i}\right)^2}\right]. \tag{8}$$

Equivalently, Eq (7) can be written as:

$$\mathscr{E}_{\text{click},nm,j_A j_B|\chi} = \frac{p_{j_A}p_{j_B}p_n^{j_A}p_m^{j_B}}{p_{k_A}p_{k_B}p_n^{k_A}p_m^{k_B}}\mathscr{E}_{\text{click},nm,k_A k_B|\chi} + \Delta_{\chi,nm}^{j_A j_B k_A k_B}, \tag{9}$$

where the parameter $\Delta_{\chi,nm}^{j_A j_B k_A k_B} \in \left[-p_{j_A}p_{j_B}p_n^{j_A}p_m^{j_B}N_\chi D_{\chi,nm}^{j_A j_B,k_A k_B},\ p_{j_A}p_{j_B}p_n^{j_A}p_m^{j_B}N_\chi D_{\chi,nm}^{j_A j_B,k_A k_B}\right]$.

Note that, by considering different values for the parameters $\{l_A, k_A, l_B, k_B\}$, one can obtain similar equations to Eq. (9) that relate the expected numbers of events corresponding to different intensity settings. These equations can be used as linear constraints to the estimation procedure.

Note that in the asymptotic limit where $N_\chi \to \infty$, we have that the actual number of events converge to the expected number of events and therefore one can directly use the constraints given by Eq. (9).

The Finite-Key Regime

In the previous section, we have derived mathematical relations between the expected numbers of events associated with different intensity settings in the asymptotic limit. By applying Azuma's inequality[7], this analysis can be extended to the realistic regime where Alice and Bob send a finite number (N) of pulses. For this, one can consider a virtual scenario in which Alice and Bob first decide the basis χ for each of the total N rounds. And thus the value of the quantity N_χ is now fixed. To be precise, in such a fictitious scenario we perform a delayed choice of the intensity setting "*after*" finishing all the basis choices in the actual protocol. Therefore, N_χ is fixed, and then we can use Azuma's inequality for the decoy-state method. Note that the Kraus operator acting on the ith pulse depends on all the previous intensity choices, all the basis choices and Eve's arbitrary operation that is dependent on all the announcements she made. Importantly, the trace distance argument is still valid thanks to the generality of the trace distance as well as the fact that Eve does not know Alice and Bob's intensity information for the ith pulse in advance. According to Azuma's inequality, we have that

$$\mathscr{E}_{\text{click},nm,j_A j_B|\chi} \equiv \sum_{i=1}^{N_\chi}\Pr^i(\text{click}, nm, j_A j_B | \chi) = N_{\text{click},nm,j_A j_B|\chi} + \delta_{\chi,nm}^{j_A j_B}, \tag{10}$$

where N_χ is the *actual* number of trials, and $N_{\text{click},nm,j_A j_B|\chi}$ is the actual number of such events. The deviation term $\delta_{\chi,nm}^{j_A j_B}$ lies in the interval $[-\Delta_{\chi,nm}^{j_A j_B}, \widehat{\Delta}_{\chi,nm}^{j_A j_B}]$ except for a small error probability $\varepsilon_{\chi,nm}^{j_A j_B} + \widehat{\varepsilon}_{\chi,nm}^{j_A j_B}$ where the bounds $\Delta_{\chi,nm}^{j_A j_B}$ and $\widehat{\Delta}_{\chi,nm}^{j_A j_B}$ are given by $\Delta_{\chi,nm}^{j_A j_B} = f(N_\chi, \varepsilon_{\chi,nm}^{j_A j_B})$ and $\widehat{\Delta}_{\chi,nm}^{j_A j_B} = f(N_\chi, \widehat{\varepsilon}_{\chi,nm}^{j_A j_B})$, respectively. The function $f(x,y)$ is given by

$$f(x,y) = \sqrt{2x\ln(1/y)}. \tag{11}$$

That is, $\varepsilon_{\chi,nm}^{j_A j_B}$ quantifies the error probability that $\delta_{\chi,nm}^{j_A j_B}$ is not lower bounded by $-\Delta_{\chi,nm}^{j_A j_B}$ and $\widehat{\varepsilon}_{\chi,nm}^{j_A j_B}$ quantifies the error probability that the parameter $\delta_{\chi,nm}^{j_A j_B}$ is not upper bounded by $\widehat{\Delta}_{\chi,nm}^{j_A j_B}$. Note that Azuma's inequality takes any correlation between the probabilities $\Pr^i(\text{click}, nm, j_A j_B | \chi)$ associated with different trials into account. To simplify our notation, here we omit, however, the explicit dependence of the probabilities $\Pr^i(\text{click}, nm, j_A j_B | \chi)$ with all the previous events, all the basis choices, and Eve's arbitrary operation that is dependent on all the announcements she made.

On the other hand, we also have that

$$\mathscr{E}_{\text{click},j_A j_B|\chi} \equiv \sum_{i=1}^{N_\chi} \Pr^i(\text{click}, j_A j_B | \chi) = N_{\text{click},j_A j_B|\chi} + \delta_\chi^{j_A j_B}, \tag{12}$$

where $\mathscr{E}_{\text{click},j_A j_B|\chi}$ denotes the expected number of events when Alice and Bob select the intensity settings γ^{j_A} and γ^{j_B}, respectively, and the relay obtains a successful measurement result given that both Alice and Bob select the χ basis in N_χ trials. $N_{\text{click},j_A j_B|\chi}$ is the corresponding actual number. The deviation term $\delta_\chi^{j_A j_B}$ lies in the interval $[-\Delta_\chi^{j_A j_B}, \widehat{\Delta}_\chi^{j_A j_B}]$ except for a small error probability $\varepsilon_\chi^{j_A j_B} + \widehat{\varepsilon}_\chi^{j_A j_B}$ where the bounds $\Delta_\chi^{j_A j_B}$ and $\widehat{\Delta}_\chi^{j_A j_B}$ are given by $\Delta_\chi^{j_A j_B} = f(N_\chi, \varepsilon_\chi^{j_A j_B})$ and $\widehat{\Delta}_\chi^{j_A j_B} = f(N_\chi, \widehat{\varepsilon}_\chi^{j_A j_B})$, respectively. That is, $\varepsilon_\chi^{j_A j_B}$ quantifies the error probability that $\delta_\chi^{j_A j_B}$ is not lower bounded by $-\Delta_\chi^{j_A j_B}$ and $\widehat{\varepsilon}_\chi^{j_A j_B}$ quantifies the error probability that the parameter $\delta_\chi^{j_A j_B}$ is not upper bounded by $\widehat{\Delta}_\chi^{j_A j_B}$.

By combining Eqs. (10) and (12), we obtain the following equation:

$$\begin{aligned} \mathscr{E}_{\text{click},j_A j_B|\chi} &= \sum_{n,m=0}^{\infty} \mathscr{E}_{\text{click},nm,j_A j_B|\chi} \\ &= \sum_{n,m=0}^{\infty} N_{\text{click},nm,j_A j_B|\chi} + \sum_{n,m=0}^{\infty} \delta_{\chi,nm}^{j_A j_B} \\ &= N_{\text{click},j_A j_B|\chi} + \delta_\chi^{j_A j_B}. \end{aligned} \tag{13}$$

That is, we have that $N_{\text{click},j_A j_B|\chi} = \sum_{n,m=0}^{\infty} N_{\text{click},nm,j_A j_B|\chi}$ and $\delta_\chi^{j_A j_B} = \sum_{n,m=0}^{\infty} \delta_{\chi,nm}^{j_A j_B}$.

The equations above relate the expected number of events to the corresponding actual numbers. These equations provide linear constraints on the quantities that we want to estimate. More precisely, by combining Eqs (9) and (13) and by taking $k_A = k_B = \text{s}$, we obtain

$$\begin{aligned} \mathscr{E}_{\text{click},j_A j_B|\chi} &= \sum_{n,m=0}^{\infty} \mathscr{E}_{\text{click},nm,j_A j_B|\chi} \\ &= \sum_{n,m=0}^{\infty} \left(\frac{p_{j_A} p_{j_B} p_n^{j_A} p_m^{j_B}}{p_\text{s} p_\text{s} p_n^\text{s} p_m^\text{s}} \mathscr{E}_{\text{click},nm,\text{ss}|\chi} + \Delta_{\chi,nm}^{j_A j_B \text{ss}} \right) \\ &= \sum_{n,m=0}^{\infty} \left(\frac{p_{j_A} p_{j_B} p_n^{j_A} p_m^{j_B}}{p_\text{s} p_\text{s} p_n^\text{s} p_m^\text{s}} \mathscr{E}_{\text{click},nm,\text{ss}|\chi} \right) + \Delta_\chi^{j_A j_B \text{ss}} \\ &= \sum_{n,m=0}^{\infty} \frac{p_{j_A} p_{j_B} p_n^{j_A} p_m^{j_B}}{p_\text{s} p_\text{s} p_n^\text{s} p_m^\text{s}} \left(N_{\text{click},nm,\text{ss}|\chi} + \delta_{\chi,nm}^{\text{ss}} \right) + \Delta_\chi^{j_A j_B \text{ss}}, \end{aligned} \tag{14}$$

where $\Delta_\chi^{j_A j_B \text{ss}} = \sum_{n,m=0}^{\infty} \Delta_{\chi,nm}^{j_A j_B \text{ss}}$.

Then, by combining Eq. (14) with Eq (12), we obtain the following linear constraints:

$$N_{\text{click},j_A j_B|\chi} = \sum_{n,m=0}^{\infty} \frac{p_{j_A} p_{j_B} p_n^{j_A} p_m^{j_B}}{p_\text{s} p_\text{s} p_n^\text{s} p_m^\text{s}} \left(N_{\text{click},nm,\text{ss}|\chi} + \delta_{\chi,nm}^{\text{ss}} \right) + \Delta_\chi^{j_A j_B \text{ss}} - \delta_\chi^{j_A j_B}. \tag{15}$$

Thus, Eq. (15) relates the actual observed quantities $N_{\text{click},j_A j_B|\chi}$ to the quantities to be estimated, $N_{\text{click},nm,\text{ss}|\chi}$.

The equations above contain an infinite number of unknown variables. To numerically estimate the quantities $N_{\text{click},00,\text{ss}|Z}^L$, $N_{\text{click},11,\text{ss}|Z}^L$ and e_{ph}^{U} by using linear programming techniques, we need to reduce the number of unknowns to a finite set. Due to the fact that $0 \le \mathscr{E}_{\text{click},nm,j_A j_B|\chi} \le N_\chi p_{j_A} p_{j_B} p_n^{j_A} p_m^{j_B}$ for all n, m and $j_A, j_B \in \{\text{s}, \text{v}, \text{w}\}$, we have that

$$\begin{aligned} \sum_{n,m=0}^{\infty} \left(\frac{p_{j_A} p_{j_B} p_n^{j_A} p_m^{j_B}}{p_\text{s} p_\text{s} p_n^\text{s} p_m^\text{s}} \mathscr{E}_{\text{click},nm,\text{ss}|\chi} \right) &\ge \sum_{n,m=0}^{S\text{cut}} \left(\frac{p_{j_A} p_{j_B} p_n^{j_A} p_m^{j_B}}{p_\text{s} p_\text{s} p_n^\text{s} p_m^\text{s}} \mathscr{E}_{\text{click},nm,\text{ss}|\chi} \right), \\ \sum_{n,m=0}^{\infty} \left(\frac{p_{j_A} p_{j_B} p_n^{j_A} p_m^{j_B}}{p_\text{s} p_\text{s} p_n^\text{s} p_m^\text{s}} \mathscr{E}_{\text{click},nm,\text{ss}|\chi} \right) &\le \sum_{n,m=0}^{S\text{cut}} \left(\frac{p_{j_A} p_{j_B} p_n^{j_A} p_m^{j_B}}{p_\text{s} p_\text{s} p_n^\text{s} p_m^\text{s}} \mathscr{E}_{\text{click},nm,\text{ss}|\chi} \right) + \sum_{n,m=S\text{cut}+1}^{\infty} \left(\frac{p_{j_A} p_{j_B} p_n^{j_A} p_m^{j_B}}{p_\text{s} p_\text{s} p_n^\text{s} p_m^\text{s}} N_\chi p_\text{s} p_\text{s} p_n^\text{s} p_m^\text{s} \right) \\ &= \sum_{n,m=0}^{S\text{cut}} \left(\frac{p_{j_A} p_{j_B} p_n^{j_A} p_m^{j_B}}{p_\text{s} p_\text{s} p_n^\text{s} p_m^\text{s}} \mathscr{E}_{\text{click},nm,\text{ss}|\chi} \right) + N_\chi p_{j_A} p_{j_B} T_{S_\text{cut}}^{j_A j_B}, \end{aligned} \tag{16}$$

where $T^{j_A j_B}_{S_{cut}} = \sum_{n,m=S_{cut}+1}^{\infty} p_n^{j_A} p_m^{j_B} = 1 - \sum_{n,m=0}^{S_{cut}} p_n^{j_A} p_m^{j_B}$ for any natural number $S_{cut} \geq 0$.

Therefore, we obtain the following constraints:

$$\begin{aligned}
&\mathscr{E}_{\text{click,ss}|\chi} \geq \sum_{n,m=0}^{S_{cut}} \mathscr{E}_{\text{click},nm,\text{ss}|\chi}, \\
&\mathscr{E}_{\text{click,ss}|\chi} \leq \sum_{n,m=0}^{S_{cut}} \mathscr{E}_{\text{click},nm,\text{ss}|\chi} + N_\chi p_s p_s T^{\text{ss}}_{S_{cut}}, \\
&\mathscr{E}_{\text{click},j_A j_B|\chi} \geq \sum_{n,m=0}^{S_{cut}} \frac{p_{j_A} p_{j_B} p_n^{j_A} p_m^{j_B}}{p_s p_s p_n^s p_m^s} \mathscr{E}_{\text{click},nm,\text{ss}|\chi} + \Delta_\chi^{j_A j_B \text{ss}}, \\
&\mathscr{E}_{\text{click},j_A j_B|\chi} \leq \sum_{n,m=0}^{S_{cut}} \frac{p_{j_A} p_{j_B} p_n^{j_A} p_m^{j_B}}{p_s p_s p_n^s p_m^s} \mathscr{E}_{\text{click},nm,\text{ss}|\chi} + N_\chi p_{j_A} p_{j_B} T^{j_A j_B}_{S_{cut}} + \Delta_\chi^{j_A j_B \text{ss}},
\end{aligned} \tag{17}$$

for any $j_A, j_B \in \{\text{s}, \text{v}, \text{w}\}$ and $j_A j_B \neq \text{ss}$. Importantly, these equations now have a finite number of unknown variables.

Finally, according to Eqs. (10) and (12) we can replace the expected values in Eq. (17) with the corresponding actual numbers plus their deviation terms. In so doing, for example, we find that the parameter $N^L_{\text{click,00,ss}|Z}$ can be estimated by using the following linear program:

$$\begin{aligned}
\min \quad & N_{\text{click,00,ss}|Z} \\
s.t. \quad & N_{\text{click,ss}|Z} \geq \sum_{n,m=0}^{S_{cut}} \left(N_{\text{click},nm,\text{ss}|Z} + \delta^{\text{ss}}_{Z,nm} \right) - \delta^{\text{ss}}_Z, \\
& N_{\text{click,ss}|Z} \leq \sum_{n,m=0}^{S_{cut}} \left(N_{\text{click},nm,\text{ss}|Z} + \delta^{\text{ss}}_{Z,nm} \right) - \delta^{\text{ss}}_Z + N_Z p_s p_s \mathrm{T}^{\text{ss}}_{S_{cut}}, \\
& N_{\text{click},j_A j_B|Z} \geq \sum_{n,m=0}^{S_{cut}} \frac{p_{j_A} p_{j_B} p_n^{j_A} p_m^{j_B}}{p_s p_s p_n^s p_m^s} \left(N_{\text{click},nm,\text{ss}|Z} + \delta^{\text{ss}}_{Z,nm} \right) - \delta_Z^{j_A j_B} + \Delta_Z^{j_A j_B \text{ss}}, \\
& N_{\text{click},j_A j_B|Z} \leq \sum_{n,m=0}^{S_{cut}} \frac{p_{j_A} p_{j_B} p_n^{j_A} p_m^{j_B}}{p_s p_s p_n^s p_m^s} \left(N_{\text{click},nm,\text{ss}|Z} + \delta^{\text{ss}}_{Z,nm} \right) - \delta_Z^{j_A j_B} + \Delta_Z^{j_A j_B \text{ss}} \\
& \qquad + N_Z p_{j_A} p_{j_B} \mathrm{T}^{j_A j_B}_{S_{cut}}, \\
& -p_{j_A} p_{j_B} N_Z \sum_{n,m=0}^{\infty} p_n^{j_A} p_m^{j_B} D^{j_A j_B,\text{ss}}_{Z,nm} \leq \Delta_Z^{j_A j_B \text{ss}} \leq p_{j_A} p_{j_B} N_Z \sum_{n,m=0}^{\infty} p_n^{j_A} p_m^{j_B} D^{j_A j_B,\text{ss}}_{Z,nm}, \\
& -\Delta_Z^{j_A j_B} \leq \delta_Z^{j_A j_B} \leq \hat{\Delta}_Z^{j_A j_B}, \; -\Delta^{\text{ss}}_Z \leq \delta^{\text{ss}}_Z \leq \hat{\Delta}^{\text{ss}}_Z, \; -\Delta^{\text{ss}}_{Z,nm} \leq \delta^{\text{ss}}_{Z,nm} \leq \hat{\Delta}^{\text{ss}}_{Z,nm},
\end{aligned} \tag{18}$$

where $j_A, j_B \in \{\text{s}, \text{v}, \text{w}\}$ and $j_A j_B \neq \text{ss}$. The unknown variables in the linear program above are: $N_{\text{click},nm,\text{ss}|Z}$, $\delta^{\text{ss}}_{Z,nm}$, δ^{ss}_Z, $\delta_Z^{j_A j_B}$, and $\Delta_Z^{j_A j_B \text{ss}}$. The calculation of the parameters $D^{j_A j_B,\text{ss}}_{Z,nm}$ is presented in Appendix A.

The solution to the linear program above is exactly $N^L_{\text{click,00,ss}|Z}$, with a total error probability

$$\varepsilon_{Z,00} = \sum_{j_A, j_B = \text{s,v,w}} \left(\varepsilon_Z^{j_A j_B} + \hat{\varepsilon}_Z^{j_A j_B} \right) + \sum_{n,m=0}^{S_{cut}} \left(\varepsilon^{\text{ss}}_{Z,nm} + \hat{\varepsilon}^{\text{ss}}_{Z,nm} \right), \tag{19}$$

where $\varepsilon_Z^{j_A j_B}$ and $\hat{\varepsilon}_Z^{j_A j_B}$ are the error probabilities associated with the estimation of the bounds on $\delta_Z^{j_A j_B}$ with $j_A, j_B \in \{\text{s}, \text{v}, \text{w}\}$. The terms $\varepsilon^{\text{ss}}_{Z,nm}$ and $\hat{\varepsilon}^{\text{ss}}_{Z,nm}$ are the error probabilities associated with the estimation of the bounds on $\delta^{\text{ss}}_{Z,nm}$.

To estimate the parameter $N^L_{\text{click,11,ss}|Z}$, one can reuse the same linear program given by Eq. (18) after replacing the objective with "min $N_{\text{click,11,ss}|Z}$".

The steps to estimate the phase error rate e^U_{ph} are as follows. First, one can redo the analysis above for those "click" events in the relay which are associated with an error (see[5,6]). That is, now we focus on the quantities $\mathscr{E}_{\text{error},nm,j_A j_B|\chi}$ which denote the expected number of events when Alice and Bob select the intensity settings γ^{j_A} and γ^{j_B} to send an n-photon pulse and an m-photon pulse, respectively, and the relay's detectors provide a click corresponding to an error given that both Alice and Bob select the χ basis. In addition, we can use the fact that $0 \leq \mathscr{E}_{\text{error},nm,j_A j_B|\chi} \leq N_\chi p_{j_A} p_{j_B} p_n^{j_A} p_m^{j_B}$ for all n, m and $j_A, j_B \in \{\text{s}, \text{v}, \text{w}\}$. In so doing, one can obtain constraints on error events which are similar to the ones given by Eq. (17). Second, with these equations as well as the constraints given by Eq. (18) but now applied to the X basis events, one can estimate a lower bound on the number of single-photon click events when both Alice and Bob select the intensity setting γ^s given that they both select the X basis, which we denote by $N^L_{\text{click,11,ss}|X}$, as well as an upper bound on the corresponding number of errors, $N^U_{\text{error,11,ss}|X}$.

Note that the derivation of $N^L_{\text{click,11,ss}|X}$ is similar to the one of $N^L_{\text{click,11,ss}|Z}$. For this, one can use the linear program used to estimate $N^L_{\text{click,11,ss}|Z}$ after replacing all the parameters and variables in the Z basis with the corresponding ones in the X basis.

Table 2. Quantum systems defined in a THA against the PM

$\lvert\Psi_Z^i\rangle_{A,E}$ ($\lvert\Psi_Z^i\rangle_{B,E}$)	The state that Alice (Bob) prepares in the Z basis together with Eve's system in the ith trial of the protocol
$\lvert\Psi_X^i\rangle_{A,E}$ ($\lvert\Psi_X^i\rangle_{B,E}$)	The state that Alice (Bob) prepares in the X basis together with Eve's system in the ith trial of the protocol
A_q (B_q)	A virtual qubit that contains Alice's (Bob's) bit value choice
A_p (B_p)	Alice's (Bob's) photonic system that is sent to the relay via a quantum channel
A_a (B_a)	An additional ancilla system stored in Alice's (Bob's) lab to account for the loss in the transmitter
E	The back-reflected light from Eve's THA

Similarly, one can further modify the program for $N^L_{\text{click},11,\text{ss}|\text{X}}$ to calculate $N^U_{\text{error},11,\text{ss}|\text{X}}$. Specifically, one can simply replace all the numbers of click events with those of error events. In addition, one replaces "min $N_{\text{click},11,\text{ss}|\text{X}}$" with "max $N_{\text{error},11,\text{ss}|\text{X}}$" to obtain an upper bound on $N_{\text{error},11,\text{ss}|\text{X}}$. Finally, given the values of $N^L_{\text{click},11,\text{ss}|\text{Z}}$, $N^L_{\text{click},11,\text{ss}|\text{X}}$ and $N^U_{\text{error},11,\text{ss}|\text{X}}$, one can use a random sampling argument to relate the number of errors in the single-photon events in the X basis to the number of phase errors associated with the single-photon events in the Z basis and thus estimate e^{U}_{ph} [8,9]. More precisely, by using Serfling's inequality[10], we obtain that

$$e^{\text{U}}_{\text{ph}} = \frac{1}{N^L_{\text{click},11,\text{ss}|\text{Z}}} \min\left\{ \left[N^L_{\text{click},11,\text{ss}|\text{Z}} \frac{N^U_{\text{error},11,\text{ss}|\text{X}}}{N^L_{\text{click},11,\text{ss}|\text{X}}} + \left(N^L_{\text{click},11,\text{ss}|\text{Z}} + N^L_{\text{click},11,\text{ss}|\text{X}}\right) \times \Upsilon\left(N^L_{\text{click},11,\text{ss}|\text{Z}}, N^L_{\text{click},11,\text{ss}|\text{X}}, \varepsilon'\right)\right], N^L_{\text{click},11,\text{ss}|\text{Z}} \right\}, \tag{20}$$

except for a failure probability

$$\varepsilon_{\text{ph},11} \le \varepsilon' + \varepsilon_{\text{X},11} + \varepsilon_{\text{E}_\text{X},11}, \tag{21}$$

where the function $\Upsilon(x,y,z)$ is defined as $\Upsilon(x,y,z) = \sqrt{(x+1)\ln(z^{-1})/[2y(x+y)]}$, and $\varepsilon_{\text{X},11}$ and $\varepsilon_{\text{E}_\text{X},11}$ are the failure probabilities corresponding to the estimation of $N^L_{\text{click},11,\text{ss}|\text{X}}$ and $N^U_{\text{error},11,\text{ss}|\text{X}}$, respectively.

THA against the phase modulator

As already mentioned in the main text, a THA against the PM might render Alice's and Bob's output states (which now also contain Eve's systems) *basis dependent*. As a result, Eve might be able to learn partial information about Alice's and Bob's basis and bit value choices each given time.

The Asymptotic Limit

To simplify the analysis, let us first consider a scenario where Alice's and Bob's light sources are both ideal single-photon sources. Also, let us assume that Alice's and Bob's basis choices are random and do not depend on the IM or on previous emitted pulses. Let $\lvert\Psi_Z^i\rangle_{A,E}$ and $\lvert\Psi_Z^i\rangle_{B,E}$ ($\lvert\Psi_X^i\rangle_{A,E}$ and $\lvert\Psi_X^i\rangle_{B,E}$) denote the states that Alice and Bob prepare (in an equivalent entanglement-based scenario) in the Z (X) basis together with Eve's system from a THA in the ith trial of the protocol. The subscripts A, B and E denote the systems of Alice, Bob and Eve, respectively. As already mentioned, here we consider a virtual entanglement scenario where each of Alice and Bob prepares a bipartite entangled state and then measures one of the two systems to actually prepare the states that are sent to the relay. More precisely, the system A (B) above contains a virtual qubit A_q (B_q) indicating Alice's (Bob's) bit value choice, and Alice's (Bob's) photonic system A_p (B_p) that is sent to the relay via the quantum channel. In addition, the system A (B) could also contain an ancilla system A_a (B_a) stored in Alice's (Bob's) lab to account for the loss in the transmitter. And Eve's system E corresponds to the back-reflected light from a THA. That is, the system $A \equiv A_qA_pA_a$ and it is similar for B. All these quantum systems are listed in Table. 2.

The phase error rate is the fictitious bit error rate that Alice and Bob would obtain if they would measure the systems A_q and B_q in the X basis, given that they prepared the states $\lvert\Psi_Z^i\rangle_{A,E}$ and $\lvert\Psi_Z^i\rangle_{B,E}$, respectively. In order to estimate the phase error rate in the presence of information leakage from the PM, we consider a fictitious protocol where we assume that Alice and Bob meet together[11] and they decide the basis choices by measuring a so-called quantum coin[12,13]. Particularly, we assume that in the ith trial of this protocol, Alice and Bob first prepare a joint state

$$\begin{aligned}\lvert\Psi^i\rangle \equiv\ & p_Z\lvert 0_Z\rangle_{A_{ba}}\lvert 0_Z\rangle_{A_c}\lvert\Psi_Z^i\rangle_{A,E}\lvert\Psi_Z^i\rangle_{B,E} + p_X\lvert 0_Z\rangle_{A_{ba}}\lvert 1_Z\rangle_{A_c}\lvert\Psi_X^i\rangle_{A,E}\lvert\Psi_X^i\rangle_{B,E} \\ & +\sqrt{p_Zp_X}\left(\lvert 1_Z\rangle_{A_{ba}}\lvert 0_Z\rangle_{A_c}\lvert\Psi_Z^i\rangle_{A,E}\lvert\Psi_X^i\rangle_{B,E} + \lvert 1_Z\rangle_{A_{ba}}\lvert 1_Z\rangle_{A_c}\lvert\Psi_X^i\rangle_{A,E}\lvert\Psi_Z^i\rangle_{B,E}\right),\end{aligned} \tag{22}$$

where

$$
\begin{aligned}
\left|\Psi^i_{\mathrm{Z}}\right\rangle_{\mathrm{A,E}} &\equiv \tfrac{1}{\sqrt{2}}\left(|0_{\mathrm{Z}}\rangle_{\mathrm{A_q}}\left|\Psi^i_{0,\mathrm{Z}}\right\rangle_{\mathrm{A',E}} + |1_{\mathrm{Z}}\rangle_{\mathrm{A_q}}\left|\Psi^i_{1,\mathrm{Z}}\right\rangle_{\mathrm{A',E}}\right),\\
\left|\Psi^i_{\mathrm{X}}\right\rangle_{\mathrm{A,E}} &\equiv \tfrac{1}{\sqrt{2}}\left(|0_{\mathrm{Z}}\rangle_{\mathrm{A_q}}\left|\Psi^i_{0,\mathrm{X}}\right\rangle_{\mathrm{A',E}} + |1_{\mathrm{Z}}\rangle_{\mathrm{A_q}}\left|\Psi^i_{1,\mathrm{X}}\right\rangle_{\mathrm{A',E}}\right),\\
\left|\Psi^i_{\mathrm{Z}}\right\rangle_{\mathrm{B,E}} &\equiv \tfrac{1}{\sqrt{2}}\left(|0_{\mathrm{Z}}\rangle_{\mathrm{B_q}}\left|\Psi^i_{0,\mathrm{Z}}\right\rangle_{\mathrm{B',E}} + |1_{\mathrm{Z}}\rangle_{\mathrm{B_q}}\left|\Psi^i_{1,\mathrm{Z}}\right\rangle_{\mathrm{B',E}}\right),\\
\left|\Psi^i_{\mathrm{X}}\right\rangle_{\mathrm{B,E}} &\equiv \tfrac{1}{\sqrt{2}}\left(|0_{\mathrm{Z}}\rangle_{\mathrm{B_q}}\left|\Psi^i_{0,\mathrm{X}}\right\rangle_{\mathrm{B',E}} + |1_{\mathrm{Z}}\rangle_{\mathrm{B_q}}\left|\Psi^i_{1,\mathrm{X}}\right\rangle_{\mathrm{B',E}}\right).
\end{aligned}
\tag{23}
$$

In Eq. (22), the first system $\mathrm{A_{ba}}$ denotes a system in Alice's hands which decides whether or not Alice's and Bob's basis choices match by measuring it in the Z basis. More precisely, if she obtains the measurement outcome corresponding to $|0_{\mathrm{Z}}\rangle_{\mathrm{A_{ba}}}$ ($|1_{\mathrm{Z}}\rangle_{\mathrm{A_{ba}}}$), then Alice's and Bob's basis choices (do not) match. The relay can perform any operation on each received signal pair from Alice and Bob to decide in which rounds there will be "click" events. For each click event, the relay then performs some measurement on the received signal pair and both Alice and Bob measure their systems $\mathrm{A_q}$ and $\mathrm{B_q}$ in the X basis. Besides, Alice selects the $\mathrm{Z_{A_c}}$ or $\mathrm{X_{A_c}}$ basis with probabilities $p_{\mathrm{Z_{A_c}}}$ and $p_{\mathrm{X_{A_c}}}$, respectively, to measure her quantum coin, denoted by the system $\mathrm{A_c}$ in Eq. (22) in the selected basis. In Eq. (23), the system $\mathrm{A'}$ is defined as $\mathrm{A'} \equiv \mathrm{A_pA_a}$ and the definition of the system $\mathrm{B'}$ is similar.

After applying the Bloch sphere bound[14] to this fictitious scenario, we obtain

$$
\begin{aligned}
&1-2\mathrm{Pr}^i\left(X_{\mathrm{A_c}}=-\,|\mathrm{click},\mathrm{sb},\mathrm{X-error},\mathrm{X_{A_c}}\right)\\
&\le 2\sqrt{\mathrm{Pr}^i\left(\mathrm{Z_{A_c}}=1\,|\mathrm{click},\mathrm{sb},\mathrm{X-error},\mathrm{Z_{A_c}}\right)\left[1-\mathrm{Pr}^i\left(\mathrm{Z_{A_c}}=1\,|\mathrm{click},\mathrm{sb},\mathrm{X-error},\mathrm{Z_{A_c}}\right)\right]},
\end{aligned}
\tag{24}
$$

and

$$
\begin{aligned}
&1-2\mathrm{Pr}^i\left(X_{\mathrm{A_c}}=-\,|\mathrm{click},\mathrm{sb},\mathrm{No\ X-error},\mathrm{X_{A_c}}\right)\\
&\le 2\sqrt{\mathrm{Pr}^i\left(\mathrm{Z_{A_c}}=1\,|\mathrm{click},\mathrm{sb},\mathrm{No\ X-error},\mathrm{Z_{A_c}}\right)\left[1-\mathrm{Pr}^i\left(\mathrm{Z_{A_c}}=1\,|\mathrm{click},\mathrm{sb},\mathrm{No\ X-error},\mathrm{Z_{A_c}}\right)\right]},
\end{aligned}
\tag{25}
$$

where $\mathrm{Pr}^i\left(X_{\mathrm{A_c}}=-\,|\mathrm{click},\mathrm{sb},\ \mathrm{X-error},\mathrm{X_{A_c}}\right)$ is the conditional probability that in the ith trial Alice's X basis measurement result on the quantum coin is '$-$' given that the relay obtains a successful result in his measurement device, Alice and Bob select the same basis (sb) for the state preparation, Bob's X basis measurement outcome on $\mathrm{B_q}$ differs from that obtained by Alice when she measures her system $\mathrm{A_q}$ in the X basis (which we call an '$\mathrm{X-error}$'), and Alice performs the $\mathrm{X_{A_c}}$ basis measurement on the quantum coin. The other conditional probabilities that appear in Eqs. (24) and (25) are defined similarly.

Then, we multiply Eq. (24) by

$$
\mathrm{Pr}^i\left(\mathrm{Z_{A_c}}\,|\mathrm{click}\right)\mathrm{Pr}^i\left(\mathrm{sb},\mathrm{X-error}\,|\mathrm{click},\mathrm{X_{A_c}}\right)=\mathrm{Pr}^i\left(\mathrm{Z_{A_c}}\,|\mathrm{click}\right)\mathrm{Pr}^i\left(\mathrm{sb},\mathrm{X-error}\,|\mathrm{click},\mathrm{Z_{A_c}}\right), \tag{26}
$$

and we multiply Eq. (25) by

$$
\mathrm{Pr}^i\left(\mathrm{Z_{A_c}}\,|\mathrm{click}\right)\mathrm{Pr}^i\left(\mathrm{sb},\mathrm{No\ X-error}\,|\mathrm{click},\mathrm{X_{A_c}}\right)=\mathrm{Pr}^i\left(\mathrm{Z_{A_c}}\,|\mathrm{click}\right)\mathrm{Pr}^i\left(\mathrm{sb},\mathrm{No\ X-error}\,|\mathrm{click},\mathrm{Z_{A_c}}\right). \tag{27}
$$

After adding both results together, we obtain

$$
\begin{aligned}
&\mathrm{Pr}^i\left(\mathrm{Z_{A_c}}\,|\mathrm{click}\right)\mathrm{Pr}^i\left(\mathrm{sb}\,|\mathrm{click},\mathrm{X_{A_c}}\right)-2\mathrm{Pr}^i\left(\mathrm{Z_{A_c}}\,|\mathrm{click}\right)\mathrm{Pr}^i\left(X_{\mathrm{A_c}}=-,\mathrm{sb}\,|\mathrm{click},\mathrm{X_{A_c}}\right)\\
&\le 2\sqrt{\mathrm{Pr}^i\left(\mathrm{Z_{A_c}}=1,\mathrm{sb},\mathrm{X-error},\mathrm{Z_{A_c}}\,|\mathrm{click}\right)\mathrm{Pr}^i\left(\mathrm{Z_{A_c}}=0,\mathrm{sb},\mathrm{X-error},\mathrm{Z_{A_c}}\,|\mathrm{click}\right)}\\
&+2\sqrt{\mathrm{Pr}^i\left(\mathrm{Z_{A_c}}=1,\mathrm{sb},\mathrm{No\ X-error},\mathrm{Z_{A_c}}\,|\mathrm{click}\right)\mathrm{Pr}^i\left(\mathrm{Z_{A_c}}=0,\mathrm{sb},\mathrm{No\ X-error},\mathrm{Z_{A_c}}\,|\mathrm{click}\right)}.
\end{aligned}
\tag{28}
$$

Note that $\mathrm{Pr}^i\left(\mathrm{Z_{A_c}}\,|\mathrm{click}\right)=p_{\mathrm{Z_{A_c}}}$ for any round 'i' and we also have $\mathrm{Pr}^i\left(\mathrm{sb}\,|\mathrm{click},\mathrm{X_{A_c}}\right)=\mathrm{Pr}^i\left(\mathrm{sb}\,|\mathrm{click}\right)$. To relate the probabilities in Eq. (28) to the expected numbers of events, we take the sum over $i\in\{1,2,\ldots,N_{\mathrm{click}}\}$, where N_{click} is the number of click events. Due to the concavity of the square root function, we have that

$$
\begin{aligned}
&p_{\mathrm{Z_{A_c}}}\sum_{i=1}^{N_{\mathrm{click}}}\mathrm{Pr}^i\left(\mathrm{sb}\,|\mathrm{click}\right)-2p_{\mathrm{Z_{A_c}}}\sum_{i=1}^{N_{\mathrm{click}}}\mathrm{Pr}^i\left(X_{\mathrm{A_c}}=-,\mathrm{sb}\,|\mathrm{click},\mathrm{X_{A_c}}\right)\\
&\le 2\sqrt{\sum_{i=1}^{N_{\mathrm{click}}}\mathrm{Pr}^i\left(\mathrm{Z_{A_c}}=1,\mathrm{sb},\mathrm{X-error},\mathrm{Z_{A_c}}\,|\mathrm{click}\right)\sum_{i=1}^{N_{\mathrm{click}}}\mathrm{Pr}^i\left(\mathrm{Z_{A_c}}=0,\mathrm{sb},\mathrm{X-error},\mathrm{Z_{A_c}}\,|\mathrm{click}\right)}\\
&+2\sqrt{\sum_{i=1}^{N_{\mathrm{click}}}\mathrm{Pr}^i\left(\mathrm{Z_{A_c}}=1,\mathrm{sb},\mathrm{No\ X-error},\mathrm{Z_{A_c}}\,|\mathrm{click}\right)}\sqrt{\sum_{i=1}^{N_{\mathrm{click}}}\mathrm{Pr}^i\left(\mathrm{Z_{A_c}}=0,\mathrm{sb},\mathrm{No\ X-error},\mathrm{Z_{A_c}}\,|\mathrm{click}\right)}.
\end{aligned}
\tag{29}
$$

In Eq. (29), we have that $\sum_{i=1}^{N_{\text{click}}} \Pr^i(\text{sb}\,|\text{click}) = \mathscr{E}_{\text{sb|click}}$, with $\mathscr{E}_{\text{sb|click}}$ being the expected number of events where Alice and Bob select the same basis given that there is a click. Also, we have that

$$\begin{aligned}\sum_{i=1}^{N_{\text{click}}} \Pr^i(X_{\text{A}_c} = -, \text{sb}\,|\text{click}, \text{X}_{\text{A}_c}) &= \sum_{i=1}^{N_{\text{click}}} \Pr^i(\text{sb}\,|\text{click}, \text{X}_{\text{A}_c}) \Pr^i(X_{\text{A}_c} = -\,|\text{click}, \text{X}_{\text{A}_c}, \text{sb}) \\ &= \sum_{i=1}^{N_{\text{click}}} \Pr^i(\text{sb}\,|\text{click}) \Pr^i(X_{\text{A}_c} = -\,|\text{click}, \text{X}_{\text{A}_c}, \text{sb}).\end{aligned} \tag{30}$$

Note that, although in the actual protocol there is no data corresponding to the event '$X_{\text{A}_c} = -$', we can still upper bound the probability $\Pr^i(X_{\text{A}_c} = -\,|\text{click}, \text{X}_{\text{A}_c}, \text{sb})$. For this, we assume a worst-case scenario where we take the maximum value of this probability in the total number N of rounds. More precisely, we have that

$$\begin{aligned}\Pr^i(X_{\text{A}_c} = -\,|\text{click}, \text{X}_{\text{A}_c}, \text{sb}) &\leq \max_{j\in\{1,2,\ldots N\}} \Pr^j(X_{\text{A}_c} = -\,|\text{X}_{\text{A}_c}, \text{sb}) \\ &= \frac{1}{2}\left\{1 - \frac{2p_{\text{Z}}p_{\text{X}}}{p_{\text{Z}}^2 + p_{\text{X}}^2} \min_{j\in\{1,2,\ldots N\}} \text{Re}\left(\left\langle \Psi_{\text{Z}}^j \middle| \Psi_{\text{X}}^j \right\rangle_{\text{A,E}} \left\langle \Psi_{\text{Z}}^j \middle| \Psi_{\text{X}}^j \right\rangle_{\text{B,E}}\right)\right\} \\ &\equiv \Delta_{X_{\text{A}_c}=-}.\end{aligned} \tag{31}$$

The detailed calculation of the probability $\Pr^j(X_{\text{A}_c} = -\,|\text{X}_{\text{A}_c}, \text{sb})$ can be found in Appendix B.

Let us denote

$$\begin{aligned}&\sum_{i=1}^{N_{\text{click}}} \Pr^i(\text{Z}_{\text{A}_c} = 1, \text{sb}, \text{X} - \text{error}, \text{Z}_{\text{A}_c}\,|\text{click}) = \mathscr{E}_{\text{X,X-error}}, \\ &\sum_{i=1}^{N_{\text{click}}} \Pr^i(\text{Z}_{\text{A}_c} = 0, \text{sb}, \text{X} - \text{error}, \text{Z}_{\text{A}_c}\,|\text{click}) = \mathscr{E}_{\text{Z,X-error}}, \\ &\sum_{i=1}^{N_{\text{click}}} \Pr^i(\text{Z}_{\text{A}_c} = 1, \text{sb}, \text{No X} - \text{error}, \text{Z}_{\text{A}_c}\,|\text{click}) = \mathscr{E}_{\text{X,No X-error}}, \\ &\sum_{i=1}^{N_{\text{click}}} \Pr^i(\text{Z}_{\text{A}_c} = 0, \text{sb}, \text{No X} - \text{error}, \text{Z}_{\text{A}_c}\,|\text{click}) = \mathscr{E}_{\text{Z,No X-error}}.\end{aligned} \tag{32}$$

Then Eq. (29) can be written as

$$p_{\text{Z}_{\text{A}_c}} \mathscr{E}_{\text{sb|click}} \left(1 - 2\Delta_{X_{\text{A}_c}=-}\right) \leq 2\sqrt{\mathscr{E}_{\text{X,X-error}} \mathscr{E}_{\text{Z,X-error}}} + 2\sqrt{\mathscr{E}_{\text{X,No X-error}} \mathscr{E}_{\text{Z,No X-error}}}. \tag{33}$$

Eq. (33) gives the mathematical relation between the expected number of events in the asymptotic limit. Next we will explain how to extend Eq. (33) to the actual protocol in the finite-key regime by applying Azuma's inequality.

The Finite-Key Regime

Here, we apply Azuma's inequality[7] again to relate the expected numbers of events to the corresponding actual numbers of events. Let N_λ denote the actual number of times that the event 'λ' occurs in N_{click} trials.

Then Eq. (33) can be rewritten as

$$\begin{aligned}&p_{\text{Z}_{\text{A}_c}} \left(N_{\text{sb|click}} - \delta_{\text{sb|click}}\right) \left(1 - 2\Delta_{X_{\text{A}_c}=-}\right) \\ &\leq 2\sqrt{\left(N_{\text{X,X-error}} + \delta_{\text{X,X-error}}\right)\left(N_{\text{Z,X-error}} + \delta_{\text{Z,X-error}}\right)} \\ &+2\sqrt{\left(N_{\text{click,X}} - N_{\text{X,X-error}} + \delta_{\text{X,No X-error}}\right)\left(N_{\text{click,Z}} - N_{\text{Z,X-error}} + \delta_{\text{Z,No X-error}}\right)}\end{aligned}, \tag{34}$$

except for an exponentially small error probability $\sum_\lambda (\varepsilon_\lambda + \hat{\varepsilon}_\lambda)$, where $\lambda \in \{(\text{sb|click}), (\text{X}, \text{X} - \text{error}), (\text{Z}, \text{X} - \text{error}), (\text{X}, \text{No X} - \text{error}), (\text{Z}, \text{No X} - \text{error})\}$. In Eq. (34), $N_{\text{click,Z(X)}}$ denotes the actual number of events when the relay obtains a successful measurement result and Alice and Bob select the Z (X) basis, *i.e.*, $N_{\text{click,Z(X)}} = N_{\text{Z(X),X-error}} + N_{\text{Z(X),No X-error}}$. Finally, $N_{\text{Z,X-error}}$ is the quantity to be estimated, *i.e.*, the actual number of phase errors.

So far we have considered that Alice and Bob have single-photon sources, however, it is straightforward to adapt the analysis above to the MDI-QKD protocol described in Sec. based on phase randomized WCPs. Since the final key is only distilled from the data associated with the signal intensity setting, we only need to consider that now all the actual numbers in Eq. (34) actually represent the single-photon contributions within Z^{ss}. More precisely, $\{N_{\text{sb|click}}, N_{\text{X,X-error}}, N_{\text{Z,X-error}}, N_{\text{click,X}}, N_{\text{click,Z}}\}$ now refer to $\{N_{\text{sb,11,ss|click}}, N_{\text{X-error,11,ss|X}}, N_{\text{X-error,11,ss|Z}}, N_{\text{click,11,ss|X}}, N_{\text{click,11,ss|Z}}\}$, where $N_{\text{sb,11,ss|click}}$ denotes the actual number of events where Alice and Bob both select the same basis and the intensity setting γ^{s} and send a single photon state given that the relay obtains a successful measurement result. This replacement is allowed because in principle Alice and Bob can perform

a quantum non-demolition measurement to know in which pulse each of them emits a single-photon, and we can apply the analysis above to such instances. As long as we do not use explicity in which instance Alice and Bob emit a single-photon, which is exactly the case in our analysis, the security follows.

Then we can rewrite Eq. (34) as follows:

$$\begin{aligned} &p_{Z_{A_c}}\left(N_{\mathrm{sb},11,\mathrm{ss}|\mathrm{click}}-\delta_{\mathrm{sb},11,\mathrm{ss}|\mathrm{click}}\right)\left(1-2\Delta_{X_{A_c}=-}\right)\\ &\leq 2\sqrt{\left(N_{\mathrm{X-error},11,\mathrm{ss}|\mathrm{X}}+\delta_{\mathrm{X-error},11,\mathrm{ss}|\mathrm{X}}\right)\left(N_{\mathrm{X-error},11,\mathrm{ss}|\mathrm{Z}}+\delta_{\mathrm{X-error},11,\mathrm{ss}|\mathrm{Z}}\right)}\\ &+2\sqrt{\left(N_{\mathrm{click},11,\mathrm{ss}|\mathrm{X}}-N_{\mathrm{X-error},11,\mathrm{ss}|\mathrm{X}}+\delta_{\mathrm{No\ X-error},11,\mathrm{ss}|\mathrm{X}}\right)}\sqrt{\left(N_{\mathrm{click},11,\mathrm{ss}|\mathrm{Z}}-N_{\mathrm{X-error},11,\mathrm{ss}|\mathrm{Z}}+\delta_{\mathrm{No\ X-error},11,\mathrm{ss}|\mathrm{Z}}\right)}. \end{aligned} \tag{35}$$

Since we have that $N_{\mathrm{sb},11,\mathrm{ss}|\mathrm{click}}=N_{\mathrm{click},11,\mathrm{ss}|\mathrm{Z}}+N_{\mathrm{click},11,\mathrm{ss}|\mathrm{X}}\geq 2\sqrt{N_{\mathrm{click},11,\mathrm{ss}|\mathrm{Z}}N_{\mathrm{click},11,\mathrm{ss}|\mathrm{X}}}$, if we divide the LHS of Eq. (35) by $N_{\mathrm{sb},11,\mathrm{ss}|\mathrm{click}}$ and RHS of Eq. (35) by $2\sqrt{N_{\mathrm{click},11,\mathrm{ss}|\mathrm{Z}}N_{\mathrm{click},11,\mathrm{ss}|\mathrm{X}}}$, respectively, then we obtain that

$$\begin{aligned} &p_{Z_{A_c}}\left(1-\frac{\delta_{\mathrm{sb},11,\mathrm{ss}|\mathrm{click}}}{N_{\mathrm{sb},11,\mathrm{ss}|\mathrm{click}}}\right)\left(1-2\Delta_{X_{A_c}=-}\right)\\ &\leq\sqrt{\frac{\left(N_{\mathrm{X-error},11,\mathrm{ss}|\mathrm{X}}+\delta_{\mathrm{X-error},11,\mathrm{ss}|\mathrm{X}}\right)}{N_{\mathrm{click},11,\mathrm{ss}|\mathrm{X}}}\frac{\left(N_{\mathrm{X-error},11,\mathrm{ss}|\mathrm{Z}}+\delta_{\mathrm{X-error},11,\mathrm{ss}|\mathrm{Z}}\right)}{N_{\mathrm{click},11,\mathrm{ss}|\mathrm{Z}}}}\\ &+\sqrt{\left(1-\frac{N_{\mathrm{No\ X-error},11,\mathrm{ss}|\mathrm{X}}-\delta_{\mathrm{No\ X-error},11,\mathrm{ss}|\mathrm{X}}}{N_{\mathrm{click},11,\mathrm{ss}|\mathrm{X}}}\right)\left(1-\frac{N_{\mathrm{No\ X-error},11,\mathrm{ss}|\mathrm{Z}}+\delta_{\mathrm{No\ X-error},11,\mathrm{ss}|\mathrm{Z}}}{N_{\mathrm{click},11,\mathrm{ss}|\mathrm{Z}}}\right)}, \end{aligned} \tag{36}$$

also holds except for a small failure probability.

To solve Eq. (36) one can use the same procedure based on the linear optimization method that we employed in the previous section to first estimate the quantities $\{N_{\mathrm{sb},11,\mathrm{ss}|\mathrm{click}},\ N_{\mathrm{X-error},11,\mathrm{ss}|\mathrm{X}},\ N_{\mathrm{click},11,\mathrm{ss}|\mathrm{X}},\ N_{\mathrm{click},11,\mathrm{ss}|\mathrm{Z}}\}$. Note that, although the relation $N_{\mathrm{sb},11,\mathrm{ss}|\mathrm{click}}=N_{\mathrm{click},11,\mathrm{ss}|\mathrm{Z}}+N_{\mathrm{click},11,\mathrm{ss}|\mathrm{X}}$ holds in terms of the actual numbers, to estimate an upper bound on the number of phase errors by using Eq. (36), we need to obtain either a lower bound or an upper bound on each of these three parameters. This means that, we need to estimate these three parameters independently. Also, we set $p_{Z_{A_c}}$ as input and search for its optimal value by using a Monte Carlo method. For each given value of $p_{Z_{A_c}}$, the only unknown variable in Eq. (36) is $N_{\mathrm{X-error},11,\mathrm{ss}|\mathrm{Z}}$, which can be numerically estimated by using the optimization toolbox of Matlab.

Parameter estimation method for the four-intensity protocol with leaky sources

Estimation of the parameters $N^L_{\mathrm{click},00,\mathrm{ss}}$ and $N^L_{\mathrm{click},11,\mathrm{ss}}$

In this section, we present the procedure to estimate the parameters $N^L_{\mathrm{click},00,\mathrm{ss}|\mathrm{Z}}$ and $N^L_{\mathrm{click},11,\mathrm{ss}|\mathrm{Z}}$ which are needed to evaluate the secret key rate formula provided in the maintext. Due to the fact that in this protocol the intensity γ^{s} is only used for the data in the Z basis, we have that $N^L_{\mathrm{click},00,\mathrm{ss}|\mathrm{Z}}\equiv N^L_{\mathrm{click},00,\mathrm{ss}}$ and $N^L_{\mathrm{click},11,\mathrm{ss}|\mathrm{Z}}\equiv N^L_{\mathrm{click},11,\mathrm{ss}}$, where $N^L_{\mathrm{click},00,\mathrm{ss}}$ ($N^L_{\mathrm{click},11,\mathrm{ss}}$) is a lower bound on the number of events where Alice and Bob both select the intensity γ^{s} and send a vacuum (single-photon) pulse, and the relay obtains a successful measurement result. In the MDI-QKD protocol introduced in[15], the data in the Z basis is used for key distillation and the data in the X basis is used for parameter estimation, and we first estimate the quantities $N^L_{\mathrm{click},00,\mathrm{vv}}$ and $N^L_{\mathrm{click},11,\mathrm{vv}}$, which correspond to the intensity γ^{v} only in the X basis. For this we use the same techniques, like in the previous section, which rely on the trace distance argument among events only in the X basis. More precisely, the quantities $N^L_{\mathrm{click},00,\mathrm{vv}}$ and $N^L_{\mathrm{click},11,\mathrm{vv}}$ can be estimated by applying similar linear programming techniques with all the sifted data in the X basis as what has been done above. This analysis is valid because we can imagine a fictitious delayed measurement scenario in which Alice and Bob determine the basis first, and then for the events where both of them use the X basis, Alice and Bob start to choose the intensity settings. Note that since this estimation only involves events in the X basis, it is not affected by the information leakage from the PM. Next, we can relate these two quantities to the parameters $N^L_{\mathrm{click},00,\mathrm{ss}}$ and $N^L_{\mathrm{click},11,\mathrm{ss}}$, respectively, by using the trace distance argument between the X and Z basis states. For example, in Eqs. (5)-(7) if we focus on the total number of events N instead of on the N_χ events where both Alice and Bob select the χ basis and take $n=m=0$, $j_{\mathrm{A}}=j_{\mathrm{B}}=\mathrm{s}$ and $k_{\mathrm{A}}=k_{\mathrm{B}}=\mathrm{v}$, we have that

$$\mathscr{E}_{\mathrm{click},00,\mathrm{ss}}=\frac{p_{\mathrm{s}}^2(p_0^{\mathrm{s}})^2}{p_{\mathrm{v}}^2(p_0^{\mathrm{v}})^2}\mathscr{E}_{\mathrm{click},00,\mathrm{vv}}+\Delta_{00}^{\mathrm{ssvv}}, \tag{37}$$

where $\mathscr{E}_{\mathrm{click},00,\mathrm{vv}}$ is the expected number of events where both Alice and Bob select the intensity setting γ^{v} and send a vacuum pulse and the relay obtains a successful event. Note that, $\mathscr{E}_{\mathrm{click},00,\mathrm{vv}}\equiv\mathscr{E}_{\mathrm{click},00,\mathrm{vv}|\mathrm{X}}$ as the intensity setting γ^{v} is only selected in the X basis. The quantity $\mathscr{E}_{\mathrm{click},00,\mathrm{ss}}$ is defined in an analogous way and it is equal to $\mathscr{E}_{\mathrm{click},00,\mathrm{ss}|\mathrm{Z}}$. The deviation term

$\Delta_{00}^{\text{ssvv}} \in \left[-p_{\text{s}}^2(p_0^{\text{s}})^2 N D_{00}^{\text{ss,vv}},\ p_{\text{s}}^2(p_0^{\text{s}})^2 N D_{00}^{\text{ss,vv}}\right]$, where

$$D_{00}^{\text{ss,vv}} = \frac{1}{N}\sum_{i=1}^{N} D_{00}^{\text{ss,vv},i} := \frac{1}{N}\sum_{i=1}^{N} \text{Tr}\left[\sqrt{\left(\rho_{00}^{\gamma^s\gamma^s,i} - \rho_{00}^{\gamma^v\gamma^v,i}\right)^2}\right] \tag{38}$$

with $\rho_{00}^{\gamma^v\gamma^v,i}$ ($\rho_{00}^{\gamma^s\gamma^s,i}$) being the normalized joint state of both Alice's and Bob's vacuum pulses when they both select the intensity setting γ^v (γ^s) together with the systems E_{a}^{A}, E_{a}^{B}, $E_{\text{p}}^{\text{A}'}$, $E_{\text{p}}^{\text{B}'}$ in the ith trial. The definitions of E_{a}^{A}, E_{a}^{B}, $E_{\text{p}}^{\text{A}'}$ and $E_{\text{p}}^{\text{B}'}$ are the same as those of Sec. . Next we apply Azuma's inequality to Eq. (37) and obtain

$$N_{\text{click,00,ss}} = \frac{p_{\text{s}}^2(p_0^{\text{s}})^2}{p_{\text{v}}^2(p_0^{\text{v}})^2}(N_{\text{click,00,vv}} + \delta_{00}^{\text{vv}}) - \delta_{00}^{\text{ss}} + \Delta_{00}^{\text{ssvv}}, \tag{39}$$

where the parameter δ_{00}^{ss} denotes the deviation term between $\mathscr{E}_{\text{click,00,ss}}$ and $N_{\text{click,00,ss}}$ and it can be bounded by $[-\Delta_{00}^{\text{ss}}, \widehat{\Delta}_{00}^{\text{ss}}]$ except for a small error probability $\varepsilon_{00}^{\text{ss}} + \widehat{\varepsilon}_{00}^{\text{ss}}$. The bounds are given by $\Delta_{00}^{\text{ss}} = f(N, \varepsilon_{00}^{\text{ss}})$ and $\widehat{\Delta}_{00}^{\text{ss}} = f(N, \widehat{\varepsilon}_{00}^{\text{ss}})$, and the parameter δ_{00}^{vv} denotes the deviation term between $\mathscr{E}_{\text{click,00,vv}}$ and $N_{\text{click,00,vv}}$ and it can be bounded by $[-\Delta_{00}^{\text{vv}}, \widehat{\Delta}_{00}^{\text{vv}}]$ except for a small error probability $\varepsilon_{00}^{\text{vv}} + \widehat{\varepsilon}_{00}^{\text{vv}}$. The bounds are given by $\Delta_{00}^{\text{vv}} = f(N, \varepsilon_{00}^{\text{vv}})$ and $\widehat{\Delta}_{00}^{\text{vv}} = f(N, \widehat{\varepsilon}_{00}^{\text{vv}})$. In this way, given the quantity $N_{\text{click,00,vv}}^{L}$, we can estimate $N_{\text{click,00,ss}}^{\text{L}}$ according to Eq. (39).

Note that, in general, one could alternatively first estimate all $N_{\text{click,00,}kl}^{L}$ for $k,l \in \{\text{v},\text{w},0\}$ by using the linear programming method. Then by performing the trace distance argument, one obtains

$$\left|\mathscr{E}_{\text{click,00,ss}} - \sum_{k,l} q_{00,kl}\mathscr{E}_{\text{click,00,}kl}\right| \leq D_{00}^{\text{ss},\Sigma kl}, \tag{40}$$

with $k,l \in \{\text{v},\text{w},0\}$, where $q_{00,kl}$ is the normalization probability given by

$$\begin{aligned} q_{00,kl} &= \frac{p_k p_0^k p_l p_0^l}{\sum\limits_{k',l' \in \{\text{v},\text{w},0\}} p_{k'} p_0^{k'} p_{l'} p_0^{l'}}, \\ D_{00}^{\text{ss},\Sigma kl} &= \frac{1}{N}\sum_{i=1}^{N} D_{00}^{\text{ss},\Sigma kl,i} := \frac{1}{N}\sum_{i=1}^{N} \text{Tr}\left[\sqrt{(\sum_{k,l} q_{00,kl}\rho_{00}^{\gamma^k\gamma^l,i} - \rho_{00}^{\gamma^s\gamma^s,i})^2}\right]. \end{aligned} \tag{41}$$

In so doing, and after applying Azuma's inequality in Eq. (40), one can relate $N_{\text{click,00,ss}}^{L}$ to all $N_{\text{click,00,}kl}^{L}$ for $k,l \in \{\text{v},\text{w},0\}$ in a similar way like Eq. (39). Finally, one can derive $N_{\text{click,00,ss}}^{L}$ from the estimations of all the quantities $N_{\text{click,00,}kl}^{L}$. However, our simulations suggest that the improvement obtained when estimating $N_{\text{click,00,ss}}^{L}$ with this general method is negligible compared to the simpler method given by Eq. (39). Thus, in the following we use Eq. (39) to obtain $N_{\text{click,00,ss}}^{L}$ from $N_{\text{click,00,vv}}^{L}$.

Similarly, if we focus on the total number of events, N, and take $n = m = 1$, $j_{\text{A}} = j_{\text{B}} = \text{s}$ and $k_{\text{A}} = k_{\text{B}} = \text{v}$ in Eqs. (5)-(7), we can estimate the quantity $N_{\text{click,11,ss}}^{\text{L}}$ by following the same procedure explained above. We omit the explicit calculations here for simplicity.

Estimation of the parameter e_{ph}^{U}

To apply the idea of the quantum coin, we need to determine the form of the joint state prepared by Alice and Bob together with Eve's systems in a virtual scenario. To be precise, we shall consider a virtual single-photon scenario, where we assume that Alice and Bob meet together and follow the procedure introduced in[11]. That is, they prepare a joint state in each round of the protocol, which has the form[11]:

$$\begin{aligned} |\Psi\rangle \equiv\ & p_{\text{Z}}|0_{\text{Z}}\rangle_{\text{A}_{\text{ba}}}|0_{\text{Z}}\rangle_{\text{A}_{\text{c}}}|\Psi_{\text{Z}}\rangle_{\text{A}_{\text{q}},\text{A}_{\text{p}},\text{A}_{\text{a}},\text{A}_{\text{int}},\text{E}_{\text{IM}},\text{E}_{\text{PM}}}|\Psi_{\text{Z}}\rangle_{\text{B}_{\text{q}},\text{B}_{\text{p}},\text{B}_{\text{a}},\text{B}_{\text{int}},\text{E}_{\text{IM}},\text{E}_{\text{PM}}} \\ & + p_{\text{X}}|0_{\text{Z}}\rangle_{\text{A}_{\text{ba}}}|1_{\text{Z}}\rangle_{\text{A}_{\text{c}}}|\Psi_{\text{X}}\rangle_{\text{A}_{\text{q}},\text{A}_{\text{p}},\text{A}_{\text{a}},\text{A}_{\text{int}},\text{E}_{\text{IM}},\text{E}_{\text{PM}}}|\Psi_{\text{X}}\rangle_{\text{B}_{\text{q}},\text{B}_{\text{p}},\text{B}_{\text{a}},\text{B}_{\text{int}},\text{E}_{\text{IM}},\text{E}_{\text{PM}}} \\ & + \sqrt{p_{\text{Z}}p_{\text{X}}}(|1_{\text{Z}}\rangle_{\text{A}_{\text{ba}}}|0_{\text{Z}}\rangle_{\text{A}_{\text{c}}}|\Psi_{\text{Z}}\rangle_{\text{A}_{\text{q}},\text{A}_{\text{p}},\text{A}_{\text{a}},\text{A}_{\text{int}},\text{E}_{\text{IM}},\text{E}_{\text{PM}}}|\Psi_{\text{X}}\rangle_{\text{B}_{\text{q}},\text{B}_{\text{p}},\text{B}_{\text{a}},\text{B}_{\text{int}},\text{E}_{\text{IM}},\text{E}_{\text{PM}}} \\ & + |1_{\text{Z}}\rangle_{\text{A}_{\text{ba}}}|1_{\text{Z}}\rangle_{\text{A}_{\text{c}}}|\Psi_{\text{X}}\rangle_{\text{A}_{\text{q}},\text{A}_{\text{p}},\text{A}_{\text{a}},\text{A}_{\text{int}},\text{E}_{\text{IM}},\text{E}_{\text{PM}}}|\Psi_{\text{Z}}\rangle_{\text{B}_{\text{q}},\text{B}_{\text{p}},\text{B}_{\text{a}},\text{B}_{\text{int}},\text{E}_{\text{IM}},\text{E}_{\text{PM}}}), \end{aligned} \tag{42}$$

where the system A_{ba} denotes a system in Alice's hands which decides whether or not Alice's and Bob's basis choices match by measuring it in the Z basis. The system A_{c} denotes Alice's quantum coin and this system determines her basis choice by measuring it in the Z basis. The system A_{q} (B_{q}) denotes a virtual qubit, which contains Alice's (Bob's) bit value choice, the system A_{p} (B_{p}) represents Alice's (Bob's) single-photon system that she (he) sends to the relay via the quantum channel, the

system A_a (B_a) denotes an ancilla system stored in Alice's (Bob's) lab to account for the loss in the transmitter, and the system A_{int} (B_{int}) denotes a virtual system which decides Alice's (Bob's) intensity setting choice. E_{IM} and E_{PM}, on the other hand, denote the systems corresponding to the back-reflected light to Eve from the IM and the PM, respectively, together with the systems E_a^A and E_a^B in Eve's hands.

In this virtual protocol, the relay can perform any operation on each received signal pair from Alice and Bob to decide in which rounds there will be 'click' events (*i.e.*, successful measurement results with the notation used in this paper). For each click event, the relay then performs some measurement on the received signal pair and both Alice and Bob measure their systems A_q and B_q in the X basis. Besides, Alice selects the Z_{A_c} or X_{A_c} basis with probabilities $p_{Z_{A_c}}$ and $p_{X_{A_c}}$, respectively, to measure her quantum coin in the selected basis.

In general, we have that the states prepared by Alice for the Z and X bases are given by

$$\begin{aligned}
|\Psi_Z\rangle_{A_q,A_p,A_{int},E_{IM},E_{PM}} &= \frac{1}{\sqrt{2}}\left(|0_Z\rangle_{A_q}|0_Z\rangle_{A_p}\left|\sqrt{\frac{I_{max}}{2}}e^{i\theta_{0,Z}}\right\rangle_{PM} + |1_Z\rangle_{A_q}|1_Z\rangle_{A_p}\left|\sqrt{\frac{I_{max}}{2}}e^{i\theta_{1,Z}}\right\rangle_{PM}\right)\\
&\quad \otimes |\Phi_{\gamma^s}\rangle_{A_{int}}\left|\frac{\beta_s}{\sqrt{2}}e^{i\theta_s}\right\rangle_{IM}\\
|\Psi_X\rangle_{A_q,A_p,A_{int},E_{IM},E_{PM}} &= \frac{1}{\sqrt{2(p_v+p_w)}}\Big[|0_X\rangle_{A_q}|0_X\rangle_{A_p}\Big(e^{i\theta_1}\sqrt{p_v}|\Phi_{\gamma^v}\rangle_{A_{int}}\left|\frac{\beta_v}{\sqrt{2}}e^{i\theta_v}\right\rangle_{IM}\\
&\quad + e^{i\theta_2}\sqrt{p_w}|\Phi_{\gamma^w}\rangle_{A_{int}}\left|\frac{\beta_w}{\sqrt{2}}e^{i\theta_w}\right\rangle_{IM}\Big)\left|\sqrt{\frac{I_{max}}{2}}e^{i\theta_{0,X}}\right\rangle_{PM}\\
&\quad + |1_X\rangle_{A_q}|1_X\rangle_{A_p}\Big(e^{i\theta_3}\sqrt{p_v}|\Phi_{\gamma^v}\rangle_{A_{int}}\left|\frac{\beta_v}{\sqrt{2}}e^{i\theta_v}\right\rangle_{IM}\\
&\quad + e^{i\theta_4}\sqrt{p_w}|\Phi_{\gamma^w}\rangle_{A_{int}}\left|\frac{\beta_w}{\sqrt{2}}e^{i\theta_w}\right\rangle_{IM}\Big)\left|\sqrt{\frac{I_{max}}{2}}e^{i\theta_{1,X}}\right\rangle_{PM}\Big],
\end{aligned} \tag{43}$$

where $|0_Z\rangle_{A_q}$, $|1_Z\rangle_{A_q}$ and $|0_X\rangle_{A_q}$, $|1_X\rangle_{A_q}$ are the states of the virtual qubits which Alice measures in the Z or X basis, respectively, to prepare a state with a particular bit value. $\left|\Phi_{\gamma^k}\right\rangle_{A_{int}}$ is the virtual state that determines the intensity setting choice k with $k \in \{s, v, w\}$. It holds that $\langle\Phi_{\gamma^v}|\Phi_{\gamma^w}\rangle_{A_{int}} = 0$. This is so because in the X basis there are various intensity settings and Alice knows which one she uses each given time. Note that, these states could also include information leakage from the bit value choice as shown in Eq. (43). Since Alice always sends a vacuum state when she selects the intensity setting γ^0, there is no single-photon contribution to the state $|\Psi_X\rangle_{A_q,A_p,A_a,A_{int},E_{IM},E_{PM}}$ from the intensity setting γ^0. Likewise, Bob's states $|\Psi_Z\rangle_{B_q,B_p,B_a,B_{int},E_{IM},E_{PM}}$ and $|\Psi_X\rangle_{B_q,B_p,B_a,B_{int},E_{IM},E_{PM}}$ are defined in a similar way like Eq. (43) by changing the subscript 'A' with 'B'. In what follows, and in order to simplify the notation, we omit the subscripts '$A_q, A_p, A_a, A_{int}, E_{IM}, E_{PM}$' of the states prepared by Alice as well as those of the states prepared by Bob; instead, we use the subscript 'A,E' for Alice and similarly for Bob.

Now we are ready to apply the quantum coin idea to this virtual scenario. By following the same procedure applied to the virtual single-photon scenario explained in Sec. , we obtain a similar expression like Eq. (36). In particular, we find that

$$\begin{aligned}
&p_{Z_{A_c}}\left(1-\frac{\delta_{sb,11,ss+vv|click}}{N_{sb,11,ss+vv|click}}\right)\left(1-2\Delta_{X_{A_c}=-}\right)\\
&\leq \sqrt{\frac{(N_{X-error,11,vv|X}+\delta_{X-error,11,vv|X})}{N_{click,11,vv|X}}\frac{(N_{X-error,11,ss|Z}+\delta_{X-error,11,ss|Z})}{N_{click,11,ss|Z}}}\\
&+\sqrt{\left(1-\frac{N_{No\ X-error,11,vv|X}-\delta_{No\ X-error,11,vv|X}}{N_{click,11,vv|X}}\right)\left(1-\frac{N_{No\ X-error,11,ss|Z}+\delta_{No\ X-error,11,ss|Z}}{N_{click,11,ss|Z}}\right)},
\end{aligned} \tag{44}$$

where $N_{11,ss+vv|click} = N_{click,11,ss} + N_{click,11,vv}$. The quantity $N_{X-error,11,ss|Z}$ is the actual number of phase errors, which can be numerically estimated by using a similar method as that explained in Sec. and $\delta_{X-error,11,ss|Z}$ is the deviation term when using Azuma's inequality to estimate it.

Note, however, that the value of $\Delta_{X_{A_c}=-}$ is now different. From Alice and Bob's point of view, the bigger the value of $\mathrm{Re}\left(\langle\Psi_Z|\Psi_X\rangle_{A,E}\langle\Psi_Z|\Psi_X\rangle_{B,E}\right)$ is, the better. Indeed, in the ideal scenario without information leakage we have that $\mathrm{Re}\left(\langle\Psi_Z|\Psi_X\rangle_{A,E}\langle\Psi_Z|\Psi_X\rangle_{B,E}\right) = 1$. In general, Alice and Bob can choose the states $|\Phi_{\gamma^s}\rangle$, $|\Phi_{\gamma^v}\rangle$ and $|\Phi_{\gamma^w}\rangle$ such that $\langle\Phi_{\gamma^v}|\Phi_{\gamma^w}\rangle = 0$ and the phases θ_1, θ_2, θ_3 and θ_4 in order to maximize the quantity $\mathrm{Re}\left(\langle\Psi_Z|\Psi_X\rangle_{A,E}\langle\Psi_Z|\Psi_X\rangle_{B,E}\right)$. On the other hand, Eve can choose the values of θ_s, θ_v, θ_w, θ_Z and θ_X to minimize $\mathrm{Re}\left(\langle\Psi_Z|\Psi_X\rangle_{A,E}\langle\Psi_Z|\Psi_X\rangle_{B,E}\right)$. To maximize the

quantity $\mathrm{Re}\left(\langle\Psi_\mathrm{Z}\,|\Psi_\mathrm{X}\rangle_{\mathrm{A,E}}\langle\Psi_\mathrm{Z}\,|\Psi_\mathrm{X}\rangle_{\mathrm{B,E}}\right)$, without loss of generality, we can choose $\theta_1=\theta_2=\theta_3=\theta_4=0$ and obtain that

$$
\begin{aligned}
&\mathrm{Re}\left(\langle\Psi_\mathrm{Z}\,|\Psi_\mathrm{X}\rangle_{\mathrm{A,E}}\langle\Psi_\mathrm{Z}\,|\Psi_\mathrm{X}\rangle_{\mathrm{B,E}}\right)\\
&=\exp\left(\tfrac{I_\mathrm{max}}{2}\right)\cos\left(-\tfrac{I_\mathrm{max}}{2}\right)\frac{\sqrt{p_\mathrm{v}}\mathrm{Re}\left(\langle\Phi_{\gamma^\mathrm{s}}\,|\Phi_{\gamma^\mathrm{v}}\rangle\left\langle\frac{\beta_\mathrm{s}e^{i\theta_\mathrm{s}}}{\sqrt{2}}\,\middle|\,\frac{\beta_\mathrm{v}e^{i\theta_\mathrm{v}}}{\sqrt{2}}\right\rangle_{\mathrm{IM}}\right)+\sqrt{p_\mathrm{w}}\mathrm{Re}\left(\langle\Phi_{\gamma^\mathrm{s}}\,|\Phi_{\gamma^\mathrm{w}}\rangle\left\langle\frac{\beta_\mathrm{s}e^{\mathrm{i}\theta_\mathrm{s}}}{\sqrt{2}}\,\middle|\,\frac{\beta_\mathrm{w}e^{\mathrm{i}\theta_\mathrm{w}}}{\sqrt{2}}\right\rangle_{\mathrm{IM}}\right)}{\sqrt{p_\mathrm{v}+p_\mathrm{w}}}\\
&=\exp\left(\tfrac{I_\mathrm{max}}{2}\right)\cos\left(-\tfrac{I_\mathrm{max}}{2}\right)\frac{p_\mathrm{v}\mathrm{Re}\left(\langle\beta_\mathrm{s}e^{i\theta_\mathrm{s}}\,|\beta_\mathrm{v}e^{i\theta_\mathrm{v}}\rangle_{\mathrm{IM}}\right)+p_\mathrm{w}\mathrm{Re}\left(\langle\beta_\mathrm{s}e^{\mathrm{i}\theta_\mathrm{s}}\,|\beta_\mathrm{w}e^{\mathrm{i}\theta_\mathrm{w}}\rangle_{\mathrm{IM}}\right)}{2(p_\mathrm{v}+p_\mathrm{w})},
\end{aligned}
\tag{45}
$$

where we have chosen $\langle\Phi_{\gamma^\mathrm{s}}\,|\Phi_{\gamma^\mathrm{v}}\rangle=\frac{\sqrt{p_\mathrm{v}}}{\sqrt{p_\mathrm{v}+p_\mathrm{w}}}$ and $\langle\Phi_{\gamma^\mathrm{s}}\,|\Phi_{\gamma^\mathrm{w}}\rangle=\frac{\sqrt{p_\mathrm{w}}}{\sqrt{p_\mathrm{v}+p_\mathrm{w}}}$.

Trace distance parameters

The three-intensity decoy-state MDI-QKD protocol

For simplicity, we assume that there is no quantum correlation between Alice's (Bob's) and Eve's systems[6]. As a result, the parameter $D_{\mathrm{Z},nm}^{j_\mathrm{A}j_\mathrm{B},\mathrm{ss}}$ does not depend on the basis Z nor on the photon number n,m. Therefore, we shall denote it by $D^{j_\mathrm{A}j_\mathrm{B},\mathrm{ss}}$. Below we show the values of $D^{j_\mathrm{A}j_\mathrm{B},\mathrm{ss}}$ for the three different cases considered in the simulation. The detailed calculations are similar to those in[5].

Case 1

In this case, $D^{j_\mathrm{A}j_\mathrm{B},\mathrm{ss}}$ is given by

$$
\begin{aligned}
D^{j_\mathrm{A}j_\mathrm{B},\mathrm{ss}}&=\sqrt{1-\left|\left\langle\beta_{j_\mathrm{A}}e^{\mathrm{i}\theta_{j_\mathrm{A}}}\,|\beta_\mathrm{s}e^{\mathrm{i}\theta_\mathrm{s}}\right\rangle\times\left\langle\beta_{j_\mathrm{B}}e^{\mathrm{i}\theta_{j_\mathrm{B}}}\,|\beta_\mathrm{s}e^{\mathrm{i}\theta_\mathrm{s}}\right\rangle\right|^2}\\
&=\sqrt{1-\exp\left\{2I_\mathrm{max}\left[\cos\left(\theta_\mathrm{s}-\theta_{j_\mathrm{A}}\right)+\cos\left(\theta_\mathrm{s}-\theta_{j_\mathrm{B}}\right)-2\right]\right\}}.
\end{aligned}
\tag{46}
$$

Case 2

In this case, we have that

$$
\begin{aligned}
D^{j_\mathrm{A}j_\mathrm{B},\mathrm{ss}}&=\sqrt{1-\left|\left\langle\beta_{j_\mathrm{A}}e^{\mathrm{i}\theta_{j_\mathrm{A}}}\,|\beta_\mathrm{s}e^{\mathrm{i}\theta_\mathrm{s}}\right\rangle\times\left\langle\beta_{j_\mathrm{B}}e^{\mathrm{i}\theta_{j_\mathrm{B}}}\,|\beta_\mathrm{s}e^{\mathrm{i}\theta_\mathrm{s}}\right\rangle\right|^2}\\
&=\{\sqrt{1-\exp\left\{\frac{I_\mathrm{max}}{\gamma^\mathrm{s}}\left[2\sqrt{\gamma^\mathrm{s}\gamma^{j_\mathrm{A}}}\cos\left(\theta_\mathrm{s}-\theta_{j_\mathrm{A}}\right)+2\sqrt{\gamma^\mathrm{s}\gamma^{j_\mathrm{B}}}\cos\left(\theta_\mathrm{s}-\theta_{j_\mathrm{B}}\right)-\gamma^{j_\mathrm{A}}-\gamma^{j_\mathrm{B}}\right]\right\}}.
\end{aligned}
\tag{47}
$$

Case 3

In this last case, we further assume that the following constraints hold: $I_\mathrm{max}\le\log 2$ and $\gamma^\mathrm{w}\le\gamma^\mathrm{v}\le\gamma^\mathrm{s}$ for any $P_\mathrm{cut}\ge 1$. According to the definition of the states, it turns out that $D^{j_\mathrm{A}j_\mathrm{B},\mathrm{ss}}$ is given by

$$
\begin{aligned}
D^{j_\mathrm{A}j_\mathrm{B},\mathrm{ss}}&=\sqrt{1-\left|\left\langle\beta_{j_\mathrm{A}}e^{\mathrm{i}\theta_{j_\mathrm{A}}}\,|\beta_\mathrm{s}e^{\mathrm{i}\theta_\mathrm{s}}\right\rangle\times\left\langle\beta_{j_\mathrm{B}}e^{\mathrm{i}\theta_{j_\mathrm{B}}}\,|\beta_\mathrm{s}e^{\mathrm{i}\theta_\mathrm{s}}\right\rangle\right|^2}\\
&=\frac{1}{2}\sum_{n,m=0}^{\infty}\left|\left[\exp\left(-\beta_{j_\mathrm{A}}^2\right)\frac{\left(\beta_{j_\mathrm{A}}\right)^{2n}}{n!}-\exp\left(-\beta_\mathrm{s}^2\right)\frac{(\beta_\mathrm{s})^{2n}}{n!}\right]\left[\exp\left(-\beta_{j_\mathrm{B}}^2\right)\frac{\left(\beta_{j_\mathrm{B}}\right)^{2m}}{m!}-\exp\left(-\beta_\mathrm{s}^2\right)\frac{(\beta_\mathrm{s})^{2m}}{m!}\right]\right|\\
&=\frac{\exp(-I_\mathrm{max})}{2}\sum_{n,m=0}^{\infty}\frac{(I_\mathrm{max})^{n+m}}{n!m!}\left|\left\{1-\exp\left[I_\mathrm{max}\left(1-\gamma^{j_\mathrm{A}}/\gamma^\mathrm{s}\right)\right]\frac{\left(\gamma^{j_\mathrm{A}}\right)^n}{(\gamma^\mathrm{s})^n}\right\}\left\{1-\exp\left[I_\mathrm{max}\left(1-\gamma^{j_\mathrm{B}}/\gamma^\mathrm{s}\right)\right]\frac{\left(\gamma^{j_\mathrm{B}}\right)^m}{(\gamma^\mathrm{s})^m}\right\}\right|\\
&\le\frac{1}{2}-\frac{\exp(-I_\mathrm{max})}{2}\sum_{n,m=0}^{P_\mathrm{cut}}\frac{(I_\mathrm{max})^{n+m}}{n!m!}\left[1-\left|1-\exp\left[I_\mathrm{max}\left(1-\frac{\gamma^{j_\mathrm{A}}}{\gamma^\mathrm{s}}\right)\right]\frac{\left(\gamma^{j_\mathrm{A}}\right)^n}{(\gamma^\mathrm{s})^n}\right|\right]\left[1-\left|1-\exp\left[I_\mathrm{max}\left(1-\frac{\gamma^{j_\mathrm{B}}}{\gamma^\mathrm{s}}\right)\right]\frac{\left(\gamma^{j_\mathrm{B}}\right)^m}{(\gamma^\mathrm{s})^m}\right|\right],
\end{aligned}
\tag{48}
$$

for any $P_\mathrm{cut}\ge 0$.

The four-intensity decoy-state MDI-QKD protocol

In this section, we present the trace distance parameters for the four-intensity MDI-QKD protocol. We make the same assumptions as those in the previous section. The only difference comes from the fact that now the back-reflected light from the PM also contributes to the trace distance parameters. In particular, we obtain the following results in the three cases.

Case 1

In this case, $D^{j_A j_B,\mathrm{ss}}$ is given by

$$\begin{aligned} D^{j_A j_B,\mathrm{ss}} &= \sqrt{1-\left|\left\langle \frac{\beta_{j_A} e^{\mathrm{i}\theta_{j_A}}}{\sqrt{2}} \middle| \frac{\beta_{\mathrm{s}} e^{\mathrm{i}\theta_{\mathrm{s}}}}{\sqrt{2}} \right\rangle \left\langle \frac{\beta_{j_B} e^{\mathrm{i}\theta_{j_B}}}{\sqrt{2}} \middle| \frac{\beta_{\mathrm{s}} e^{\mathrm{i}\theta_{\mathrm{s}}}}{\sqrt{2}} \right\rangle \left(\left\langle \sqrt{\frac{I_{\max}}{2}} e^{\mathrm{i}\theta_{\mathrm{X}}} \middle| \sqrt{\frac{I_{\max}}{2}} e^{\mathrm{i}\theta_{\mathrm{Z}}} \right\rangle\right)^2 \right|^2} \\ &= \sqrt{1-\exp\left\{\tfrac{1}{8} I_{\max}\left[\cos\left(\theta_{\mathrm{s}}-\theta_{j_A}\right)+\cos\left(\theta_{\mathrm{s}}-\theta_{j_B}\right)+2\cos\left(\theta_{\mathrm{Z}}-\theta_{\mathrm{X}}\right)-4\right]\right\}}. \end{aligned} \tag{49}$$

Case 2

In this case, we have that

$$\begin{aligned} D^{j_A j_B,\mathrm{ss}} &= \sqrt{1-\left|\left\langle \frac{\beta_{j_A} e^{\mathrm{i}\theta_{j_A}}}{\sqrt{2}} \middle| \frac{\beta_{\mathrm{s}} e^{\mathrm{i}\theta_{\mathrm{s}}}}{\sqrt{2}} \right\rangle \left\langle \frac{\beta_{j_B} e^{\mathrm{i}\theta_{j_B}}}{\sqrt{2}} \middle| \frac{\beta_{\mathrm{s}} e^{\mathrm{i}\theta_{\mathrm{s}}}}{\sqrt{2}} \right\rangle \left(\left\langle \sqrt{\frac{I_{\max}}{2}} e^{\mathrm{i}\theta_{\mathrm{X}}} \middle| \sqrt{\frac{I_{\max}}{2}} e^{\mathrm{i}\theta_{\mathrm{Z}}} \right\rangle\right)^2 \right|^2} \\ &= \sqrt{1-\exp\left\{\tfrac{I_{\max}}{8\gamma^{\mathrm{s}}}\Theta(\gamma^{\mathrm{s}},\gamma^{j_A},\gamma^{j_B},\theta_{\mathrm{Z}},\theta_{\mathrm{X}})\right\}}, \end{aligned} \tag{50}$$

where

$$\Theta(\gamma^{\mathrm{s}},\gamma^{j_A},\gamma^{j_B},\theta_{\mathrm{Z}},\theta_{\mathrm{X}}) = \sqrt{\gamma^{\mathrm{s}}\gamma^{j_A}}\cos\left(\theta_{\mathrm{s}}-\theta_{j_A}\right)+\sqrt{\gamma^{\mathrm{s}}\gamma^{j_B}}\cos\left(\theta_{\mathrm{s}}-\theta_{j_B}\right)+2\gamma^{\mathrm{s}}\cos\left(\theta_{\mathrm{Z}}-\theta_{\mathrm{X}}\right)-\gamma^{j_A}-\gamma^{j_B}-2. \tag{51}$$

Case 3

In this last case, we further assume that the following constraints hold: $I_{\max} \leq \log 2$ and $0 < \gamma^{\mathrm{w}} \leq \gamma^{\mathrm{v}} \leq \gamma^{\mathrm{s}}$ for any $P_{\mathrm{cut}} \geq 1$. According to the definition of the states given by Eq. (**??**), it turns out that $D^{j_A j_B,\mathrm{ss}}$ is given by

$$\begin{aligned} D^{j_A j_B,\mathrm{ss}} &= \sqrt{1-\left|\left\langle \frac{\beta_{j_A} e^{\mathrm{i}\theta_{j_A}}}{\sqrt{2}} \middle| \frac{\beta_{\mathrm{s}} e^{\mathrm{i}\theta_{\mathrm{s}}}}{\sqrt{2}} \right\rangle \left\langle \frac{\beta_{j_B} e^{\mathrm{i}\theta_{j_B}}}{\sqrt{2}} \middle| \frac{\beta_{\mathrm{s}} e^{\mathrm{i}\theta_{\mathrm{s}}}}{\sqrt{2}} \right\rangle \left(\left\langle \sqrt{\frac{I_{\max}}{2}} e^{\mathrm{i}\theta_{\mathrm{X}}} \middle| \sqrt{\frac{I_{\max}}{2}} e^{\mathrm{i}\theta_{\mathrm{Z}}} \right\rangle\right)^2 \right|^2} \\ &= \frac{1}{2}\sum_{n,m=0}^{\infty}\left|\left\{\exp\left[-\frac{1}{2}\left(\beta_{j_A}^2+I_{\max}\right)\right]\frac{\left(\beta_{j_A}^2+I_{\max}\right)^n}{n!}-\exp\left(-\beta_{\mathrm{s}}^2\right)\frac{(\beta_{\mathrm{s}})^{2n}}{n!}\right\}\right. \\ &\quad \left.\left\{\exp\left[-\frac{1}{2}\left(\beta_{j_B}^2+I_{\max}\right)\right]\frac{\left(\beta_{j_B}^2+I_{\max}\right)^m}{m!}-\exp\left(-\beta_{\mathrm{s}}^2\right)\frac{(\beta_{\mathrm{s}})^{2m}}{m!}\right\}\right| \\ &= \frac{\exp(-I_{\max}/2)}{2}\sum_{n,m=0}^{\infty}\frac{(I_{\max})^{n+m}}{n!m!}\left|\left\{1-\exp\left[I_{\max}\left(1-\frac{\gamma^{j_A}+\gamma^{\mathrm{s}}}{2\gamma^{\mathrm{s}}}\right)\right]\frac{\left(\gamma^{j_A}+\gamma^{\mathrm{s}}\right)^n}{(2\gamma^{\mathrm{s}})^n}\right\}\left\{1-\exp\left[I_{\max}\left(1-\frac{\gamma^{j_B}+\gamma^{\mathrm{s}}}{2\gamma^{\mathrm{s}}}\right)\right]\frac{\left(\gamma^{j_B}+\gamma^{\mathrm{s}}\right)^m}{(2\gamma^{\mathrm{s}})^m}\right\}\right| \\ &\leq \frac{1}{2}-\frac{\exp(-I_{\max}/2)}{2}\sum_{n,m=0}^{P_{\mathrm{cut}}}\frac{(I_{\max})^{n+m}}{n!m!}\left[1-\left|1-\exp\left[I_{\max}\left(1-\frac{\gamma^{j_A}+\gamma^{\mathrm{s}}}{2\gamma^{\mathrm{s}}}\right)\right]\frac{\left(\gamma^{j_A}+\gamma^{\mathrm{s}}\right)^n}{(2\gamma^{\mathrm{s}})^n}\right|\right] \\ &\quad \times\left[1-\left|1-\exp\left[I_{\max}\left(1-\frac{\gamma^{j_B}+\gamma^{\mathrm{s}}}{2\gamma^{\mathrm{s}}}\right)\right]\frac{\left(\gamma^{j_B}+\gamma^{\mathrm{s}}\right)^m}{(2\gamma^{\mathrm{s}})^m}\right|\right], \end{aligned} \tag{52}$$

for any $P_{\mathrm{cut}} \geq 0$.

Calculation of the probability $\Pr^j\left(X_{A_c} = - \middle| X_{A_c}, \mathrm{sb}\right)$

According to Eq. (22), if we consider the basis matched case, the normalized state has the form:

$$\begin{aligned} \left|\Psi^j\right\rangle &= \left(\frac{p_{\mathrm{Z}}}{\sqrt{p_{\mathrm{Z}}^2+p_{\mathrm{X}}^2}}|0_{\mathrm{Z}}\rangle_{A_c}\left|\Psi_{\mathrm{Z}}^j\right\rangle_{A,E}\left|\Psi_{\mathrm{Z}}^j\right\rangle_{B,E}+\frac{p_{\mathrm{X}}}{\sqrt{p_{\mathrm{Z}}^2+p_{\mathrm{X}}^2}}|1_{\mathrm{Z}}\rangle_{A_c}\left|\Psi_{\mathrm{X}}^j\right\rangle_{A,E}\left|\Psi_{\mathrm{X}}^j\right\rangle_{B,E}\right)|0_{\mathrm{Z}}\rangle_{A_{ba}} \\ &= \left(\frac{p_{\mathrm{Z}}}{\sqrt{p_{\mathrm{Z}}^2+p_{\mathrm{X}}^2}}\frac{|+\rangle_{A_c}+|-\rangle_{A_c}}{\sqrt{2}}\left|\Psi_{\mathrm{Z}}^j\right\rangle_{A,E}\left|\Psi_{\mathrm{Z}}^j\right\rangle_{B,E}+\frac{p_{\mathrm{X}}}{\sqrt{p_{\mathrm{Z}}^2+p_{\mathrm{X}}^2}}\frac{|+\rangle_{A_c}-|-\rangle_{A_c}}{\sqrt{2}}|1_{\mathrm{Z}}\rangle_{A_c}\left|\Psi_{\mathrm{X}}^j\right\rangle_{A,E}\left|\Psi_{\mathrm{X}}^j\right\rangle_{B,E}\right)|0_{\mathrm{Z}}\rangle_{A_{ba}} \\ &= \left[\frac{p_{\mathrm{Z}}}{\sqrt{2\left(p_{\mathrm{Z}}^2+p_{\mathrm{X}}^2\right)}}\left|\Psi_{\mathrm{Z}}^j\right\rangle_{A,E}\left|\Psi_{\mathrm{Z}}^j\right\rangle_{B,E}+\frac{p_{\mathrm{X}}}{\sqrt{2\left(p_{\mathrm{Z}}^2+p_{\mathrm{X}}^2\right)}}\left|\Psi_{\mathrm{X}}^j\right\rangle_{A,E}\left|\Psi_{\mathrm{X}}^j\right\rangle_{B,E}\right]|+\rangle_{A_c}|0_{\mathrm{Z}}\rangle_{A_{ba}} \\ &\quad +\left[\frac{p_{\mathrm{Z}}}{\sqrt{2\left(p_{\mathrm{Z}}^2+p_{\mathrm{X}}^2\right)}}\left|\Psi_{\mathrm{Z}}^j\right\rangle_{A,E}\left|\Psi_{\mathrm{Z}}^j\right\rangle_{B,E}-\frac{p_{\mathrm{X}}}{\sqrt{2\left(p_{\mathrm{Z}}^2+p_{\mathrm{X}}^2\right)}}\left|\Psi_{\mathrm{X}}^j\right\rangle_{A,E}\left|\Psi_{\mathrm{X}}^j\right\rangle_{B,E}\right]|-\rangle_{A_c}|0_{\mathrm{Z}}\rangle_{A_{ba}}, \end{aligned} \tag{53}$$

where $|+\rangle_{\mathrm{A_c}}$ and $|-\rangle_{\mathrm{A_c}}$ are the two eigenstates of the quantum coin in the $\mathrm{X_{A_c}}$ basis with $|+\rangle_{\mathrm{A_c}} = \frac{|0_\mathrm{Z}\rangle_{\mathrm{A_c}}+|1_\mathrm{Z}\rangle_{\mathrm{A_c}}}{\sqrt{2}}$ and $|-\rangle_{\mathrm{A_c}} = \frac{|0_\mathrm{Z}\rangle_{\mathrm{A_c}}-|1_\mathrm{Z}\rangle_{\mathrm{A_c}}}{\sqrt{2}}$.

In each round, the conditional probability $\Pr^j\left(X_{\mathrm{A_c}} = - | \mathrm{X_{A_c}}, \mathrm{sb}\right)$ is given by

$$\begin{aligned}
&\Pr^j\left(X_{\mathrm{A_c}} = - | \mathrm{X_{A_c}}, \mathrm{sb}\right) \\
&= \left[\tfrac{(p_\mathrm{Z})^2}{2(p_\mathrm{Z}^2+p_\mathrm{X}^2)}\left\langle\Psi_\mathrm{Z}^j|\Psi_\mathrm{Z}^j\right\rangle_{\mathrm{A,E}}\left\langle\Psi_\mathrm{Z}^j|\Psi_\mathrm{Z}^j\right\rangle_{\mathrm{B,E}} + \tfrac{(p_\mathrm{X})^2}{2(p_\mathrm{Z}^2+p_\mathrm{X}^2)}\left\langle\Psi_\mathrm{X}^j|\Psi_\mathrm{X}^j\right\rangle_{\mathrm{A,E}}\left\langle\Psi_\mathrm{X}^j|\Psi_\mathrm{X}^j\right\rangle_{\mathrm{B,E}} - \tfrac{2p_\mathrm{Z}p_\mathrm{X}}{(p_\mathrm{Z}^2+p_\mathrm{X}^2)}\mathrm{Re}\left(\left\langle\Psi_\mathrm{Z}^j|\Psi_\mathrm{X}^j\right\rangle_{\mathrm{A,E}}\left\langle\Psi_\mathrm{Z}^j|\Psi_\mathrm{X}^j\right\rangle_{\mathrm{B,E}}\right)\right] \\
&= \tfrac{1}{2}\left[1 - \tfrac{2p_\mathrm{Z}p_\mathrm{X}}{p_\mathrm{Z}^2+p_\mathrm{X}^2}\mathrm{Re}\left(\left\langle\Psi_\mathrm{Z}^j|\Psi_\mathrm{X}^j\right\rangle_{\mathrm{A,E}}\left\langle\Psi_\mathrm{Z}^j|\Psi_\mathrm{X}^j\right\rangle_{\mathrm{B,E}}\right)\right].
\end{aligned} \tag{54}$$

Then we have that

$$\max_{j\in\{1,2,\ldots N\}} \Pr^j\left(X_{\mathrm{A_c}} = - | \mathrm{X_{A_c}}, \mathrm{sb}\right) = \frac{1}{2}\left\{1 - \frac{2p_\mathrm{Z}p_\mathrm{X}}{p_\mathrm{Z}^2+p_\mathrm{X}^2}\min_{j\in\{1,2,\ldots N\}}\mathrm{Re}\left(\left\langle\Psi_\mathrm{Z}^j|\Psi_\mathrm{X}^j\right\rangle_{\mathrm{A,E}}\left\langle\Psi_\mathrm{Z}^j|\Psi_\mathrm{X}^j\right\rangle_{\mathrm{B,E}}\right)\right\}. \tag{55}$$

References

1. Pereira, M., Curty, M. & Tamaki, K. Quantum key distribution with flawed and leaky sources. *npj Quantum Information* **5**, 14 (2019).

2. Pereira, M., Kato, G., Mizutani, A., Curty, M. & Tamaki, K. Quantum key distribution with correlated sources. *arXiv preprint arXiv:1908.08261* (2019).

3. Tamaki, K., Curty, M., Kato, G., Lo, H.-K. & Azuma, K. Loss-tolerant quantum cryptography with imperfect sources. *Physical Review A* **90**, 052314 (2014).

4. Nielsen, M. A. & Chuang, I. L. Quantum information and quantum computation. *Cambridge: Cambridge University Press* (2000).

5. Tamaki, K., Curty, M. & Lucamarini, M. Decoy-state quantum key distribution with a leaky source. *New Journal of Physics* **18**, 065008 (2016).

6. Wang, W., Tamaki, K. & Curty, M. Finite-key security analysis for quantum key distribution with leaky sources. *New Journal of Physics* **20**, 083027 (2018).

7. Azuma, K. Weighted sums of certain dependent random variables. *Tohoku Mathematical Journal* **19**, 357–367 (1967).

8. Tomamichel, M., Lim, C. C. W., Gisin, N. & Renner, R. Tight finite-key analysis for quantum cryptography. *Nature Communications* **3**, 634 (2012).

9. Curty, M. *et al.* Finite-key analysis for measurement-device-independent quantum key distribution. *Nature Communications* **5**, 3732 (2014).

10. Serfling, R. J. Probability inequalities for the sum in sampling without replacement. *The Annals of Statistics* **2**, 39–48 (1974).

11. Tamaki, K., Lo, H.-K., Fung, C.-H. F. & Qi, B. Phase encoding schemes for measurement-device-independent quantum key distribution with basis-dependent flaw. *Physical Review A* **85**, 042307 (2012).

12. Gottesman, D., Lo, H.-K., Lütkenhaus, N. & Preskill, J. Security of quantum key distribution with imperfect devices. *Quantum Information & Computation* **5** (2004).

13. Koashi, M. Simple security proof of quantum key distribution based on complementarity. *New Journal of Physics* **11**, 045018 (2009).

14. Tamaki, K., Koashi, M. & Imoto, N. Unconditionally secure key distribution based on two nonorthogonal states. *Physical Review Letters* **90**, 167904 (2003).

15. Zhou, Y.-H., Yu, Z.-W. & Wang, X.-B. Making the decoy-state measurement-device-independent quantum key distribution practically useful. *Physical Review A* **93**, 042324 (2016).

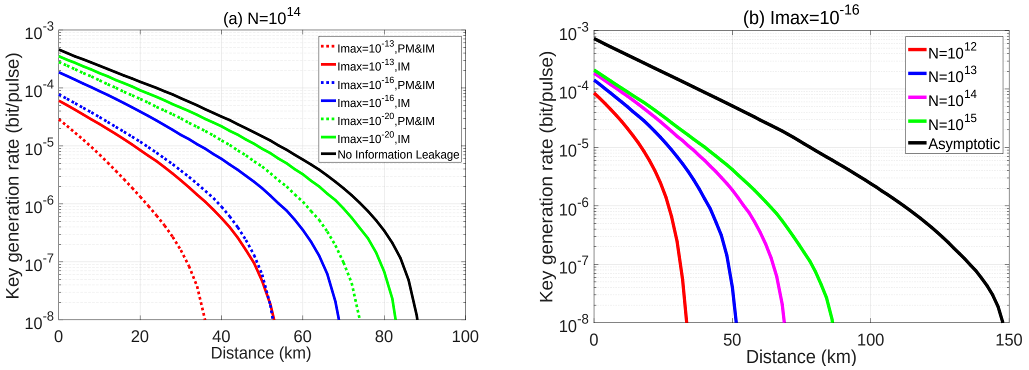

Figure 1. The secret key rate in logarithmic scale as a function of the distance in Case 2 for the three-intensity protocol. (a) Here we consider a fixed value of the total number of transmitted pulses, $N = 10^{14}$ and various values for the intensity $I_{\max}$. (b) Here we fix $I_{\max} = 10^{-16}$ and consider various values for N. Moreover, we evaluate information leakage from the IM only.

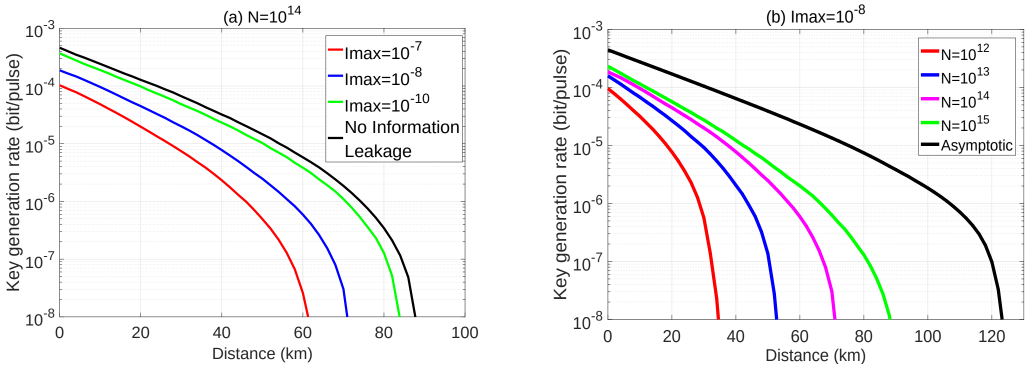

Figure 2. The secret key rate in logarithmic scale as a function of the distance in Case 3 for the three-intensity protocol. (a) Here we consider a fixed value of the total number of transmitted pulses, $N = 10^{14}$ and various values for the intensity $I_{\max}$. (b) Here we fix $I_{\max} = 10^{-8}$ and consider various values for N. Moreover, we evaluate information leakage from the IM only.

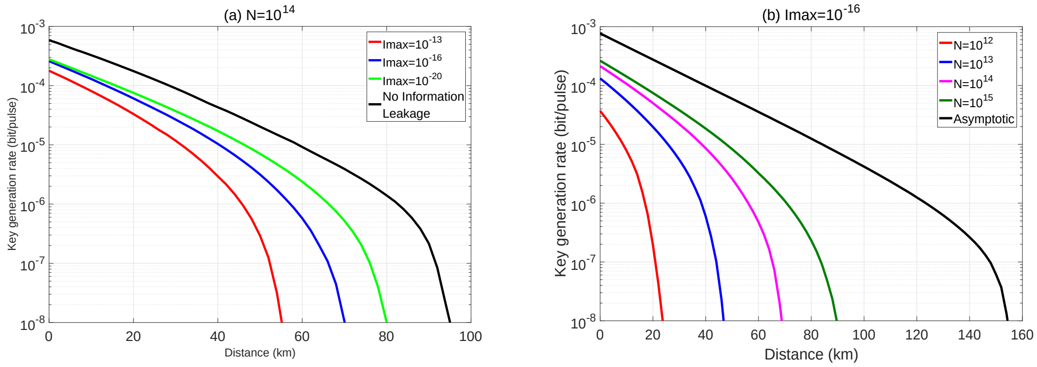

Figure 3. The secret key rate in logarithmic scale as a function of the distance in Case 2 for the four-intensity protocol. (a) Here we consider a fixed value of the total number of transmitted pulses, $N = 10^{14}$ and various values for the intensity $I_{\max}$. (b) Here we fix $I_{\max} = 10^{-16}$ and consider various values for N.

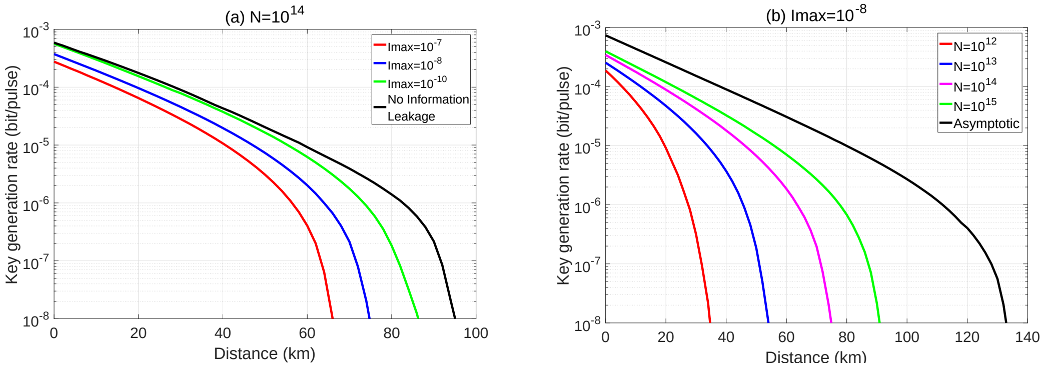

Figure 4. The secret key rate in logarithmic scale as a function of the distance in Case 3 for the four-intensity protocol. (a) Here we consider a fixed value of the total number of transmitted pulses, $N = 10^{14}$ and various values for the intensity $I_{\max}$. (b) Here we fix $I_{\max} = 10^{-8}$ and consider various values for N.
